# A Systematic Review and Meta-Analysis of the Prevalence of Congenital Myopathy

**DOI:** 10.3389/fneur.2021.761636

**Published:** 2021-11-02

**Authors:** Kun Huang, Fang-Fang Bi, Huan Yang

**Affiliations:** ^1^Department of Neurology, Xiangya Hospital, Central South University, Changsha, China; ^2^Institute of Molecular Precision Medicine and Hunan Key Laboratory of Molecular Precision Medicine, Xiangya Hospital, Central South University, Changsha, China

**Keywords:** prevalence, congenital myopathy, nemaline myopathy, core myopathy, centronuclear myopathy, congenital fiber-type disproportion myopathy

## Abstract

**Background:** Congenital myopathy constitutes a heterogeneous group of orphan diseases that are mainly classified on the basis of muscle biopsy findings. This study aims to estimate the prevalence of congenital myopathy through a systematic review and meta-analysis of the literature.

**Methods:** The PubMed, MEDLINE, Web of Science, and Cochrane Library databases were searched for original research articles published in English prior to July 30, 2021. The quality of the included studies was assessed by a checklist adapted from STrengthening the Reporting of OBservational studies in Epidemiology (STROBE). To derive the pooled epidemiological prevalence estimates, a meta-analysis was performed using the random effects model. Heterogeneity was assessed using the Cochrane *Q* statistic as well as the *I*^2^ statistic.

**Results:** A total of 11 studies were included in the systematic review and meta-analysis. Of the 11 studies included, 10 (90.9%) were considered medium-quality, one (9.1%) was considered low-quality, and no study was assessed as having a high overall quality. The pooled prevalence of congenital myopathy in the all-age population was 1.62 (95% CI, 1.13–2.11) per 100,000, while the prevalence in the child population was 2.76 (95% CI, 1.34–4.18) per 100,000. In the pediatric population, the prevalence among males was 2.92 (95% CI, −1.70 to 7.55) per 100,000, while the prevalence among females was 2.47 (95% CI, −1.67 to 6.61) per 100,000. The prevalence estimates of the all-age population per 100,000 were 0.20 (95% CI 0.10–0.35) for nemaline myopathy, 0.37 (95% CI 0.21–0.53) for core myopathy, 0.08 (95% CI −0.01 to 0.18) for centronuclear myopathy, 0.23 (95% CI 0.04–0.42) for congenital fiber-type disproportion myopathy, and 0.34 (95% CI, 0.24–0.44) for unspecified congenital myopathies. In addition, the prevalence estimates of the pediatric population per 100,000 were 0.22 (95% CI 0.03–0.40) for nemaline myopathy, 0.46 (95% CI 0.03–0.90) for core myopathy, 0.44 (95% CI 0.03–0.84) for centronuclear myopathy, 0.25 (95% CI −0.05 to 0.54) for congenital fiber-type disproportion myopathy, and 2.63 (95% CI 1.64–3.62) for unspecified congenital myopathies.

**Conclusions:** Accurate estimates of the prevalence of congenital myopathy are fundamental to supporting public health decision-making. The high heterogeneity and the lack of high-quality studies highlight the need to conduct higher-quality studies on orphan diseases.

## Introduction

Congenital myopathy is a diverse group of clinically and histologically heterogeneous muscular disorders (1). Generally, the onset occurs in the neonatal period. The diagnosis of congenital myopathy should be based on a careful review of the clinical features and confirmed by additional investigations, with an exclusionary diagnosis of other myopathies (2, 3). Previously, histopathologically oriented classification was widely used for the diagnosis of congenital myopathy, which, although still in use, tends to be replaced by genetic diagnosis in the golden era of modern genetics (4–6). Accordingly, congenital myopathy can be divided into the following several forms: nemaline myopathy, core myopathy (central core myopathy and multi-minicore myopathy), centronuclear myopathy (myotubular myopathy), congenital fiber-type disproportion myopathy, and other congenital myopathies (4).

Nemaline myopathy is characterized by the presence of small rod-like inclusions in muscle fibers (7, 8). These inclusions are clearly visualized by Gomori trichrome staining, which is mainly made up of alpha-actin and Z-band filaments (9). Core myopathy is a clinically and genetically diverse group of congenital myopathies, including central core and multi-minicore myopathies, with histopathological features of focally reduced oxidative activity on muscle biopsy (10, 11). Centronuclear myopathy is histopathologically characterized by numerous centrally placed or internalized nuclei on muscle biopsy, with the absence of necrosis or excessive regeneration (12). In congenital fiber-type disproportion myopathy, the main histological abnormality is a disproportionate difference in fiber caliber between type I and type II muscle fibers, in which type I muscle fibers are smaller than type II muscle fibers (13).

Basic prevalence information plays an indispensable role in quick and correct identification, diagnosis, and control of disease complications (14). One fundamental goal of meta-analysis, which results from the combination of existing studies, is to increase the numbers of samples and studies and to reduce the differences between the available parameters and the confidence interval (CI), which eventually leads to an argument or problem, especially in the field of medicine (15). The purpose of this study was to systematically evaluate the prevalence of congenital myopathy.

## Materials and Methods

### Search Strategy

**Figure 1:**
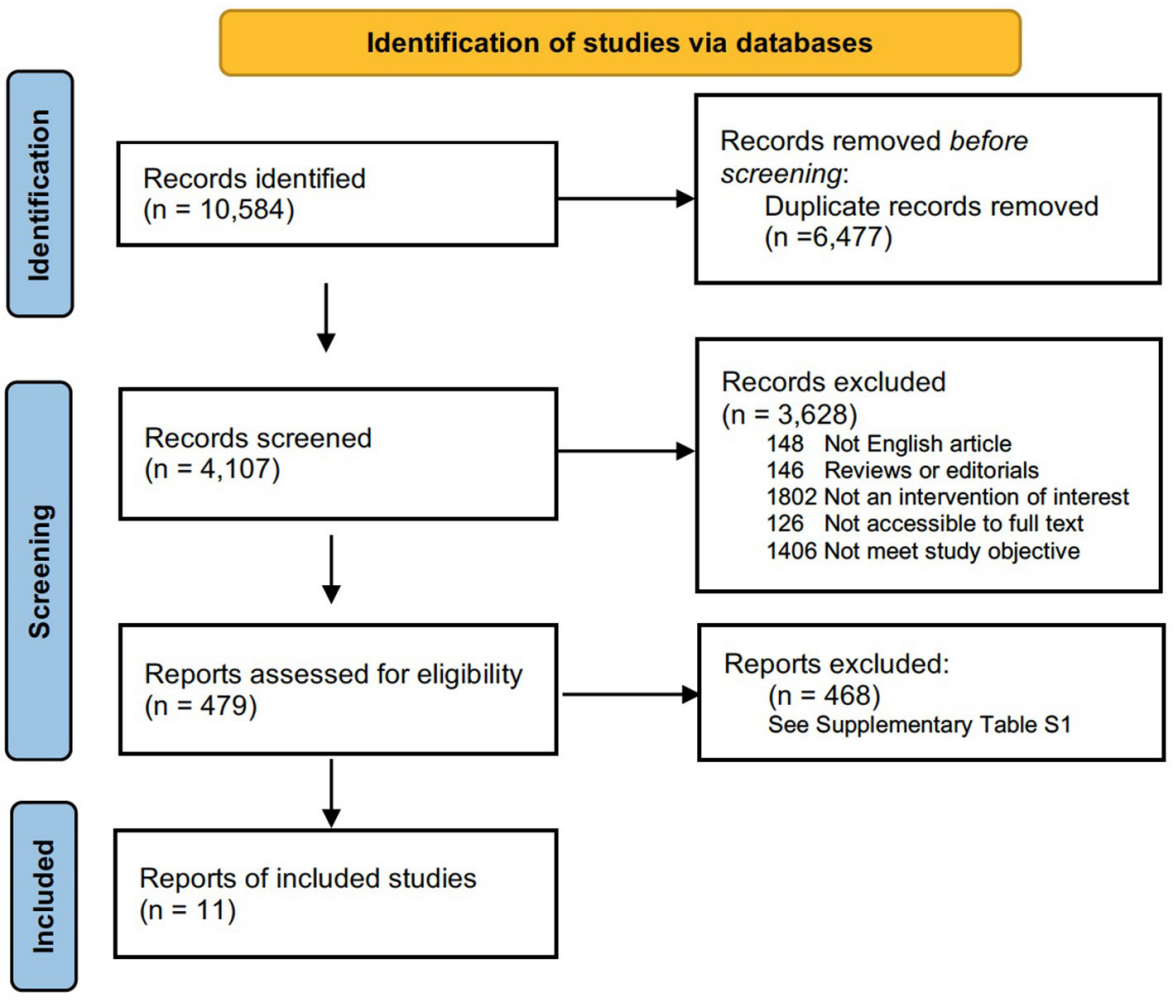
Flow chart presenting the process of study selection for systematic reviews and meta-analysis.

The search strategy used was modified from previous study (16). The literature search was restricted to articles published in English. Two authors (K.H. and FF.B.) independently searched the PubMed (1966–2021), MEDLINE (1950–2021), Web of Science (1864–2021), and Cochrane Library (2021) databases. The search strategy in PubMed was as follows: ([(congenital myopathy) OR (genetic muscle disease) OR (nemaline) OR (core myopathy) OR (centronuclear myopathy) OR (congenital fiber-type disproportion myopathy)] AND [(epidemiology) OR (prevalence)]). This retrieval also works for the other three databases. The most recent search was performed on July 30, 2021. In addition, a manual search was carried out to identify references in the identified studies to identify possible other studies. This meta-analysis followed the guidelines recommended by the PRISMA statement (Preferred Reporting Items for Systematic reviews and Meta-Analysis) (17). The PRISMA chart for the search strategy is shown in Figure 1. The studies were read thoroughly to assess the eligibility to be included in the meta-analysis.

### Inclusion and Exclusion Criteria for the Literature

Only English-written original studies that reported a numerical and well-defined measure of congenital myopathy epidemiology, such as prevalence or occurrence, birth prevalence and/or incidence, were included. Briefly, articles were included in our review if they (1) presented data on the prevalence, (2) clearly specified how many cases were diagnosed and the total population involved, and (3) established a certain diagnosis of congenital myopathy through muscle pathology or gene mutations. No restrictions regarding age, gender, ethnicity, or geography were imposed. Narrative or systematic reviews, meta-analyses, book chapters, editorials, and personal opinions were not included; however, the reference lists of reviews and meta-analyses were screened to potentially identify further studies to include.

### Data Extraction

The following data were extracted: authors, year of publication, geographic zone, data source (administrative databases, hospital and clinics medical reviews, surveys and other registries), study population (all living individuals, patients, and newborns), study period (the year at which prevalence was measured), congenital myopathy definition (ascertained by clinical examination, muscle biopsy, and genetic screening), and prevalence estimates. Original authors were contacted when further clarification and additional data were necessary. All measures of the prevalence of congenital myopathy identified in the articles were classified as either overall or child prevalence. All studies reporting the prevalence or epidemiology of congenital myopathy were carefully reviewed. Two authors (K.H. and FF-B) independently screened the titles and abstracts of all records identified by the search strategy for potential inclusion in the review. Afterward, full-text copies of articles deemed potentially relevant were retrieved, and their eligibility was assessed. Any disagreements were discussed until we reached a consensus.

### Quality Assessment of Individual Studies

To assess the quality of reporting of the published studies, the adapted STrengthening the Reporting of Observational Studies in Epidemiology (STROBE) guidelines were used in our study (see Supplementary Material 2). The adapted STROBE was modified from 22 elaborate items of STROBE (18) by selecting the five essential items most relevant to rare diseases, which is more frequently used in the research of orphan diseases (19, 20). The quality of the included studies was independently assessed by all the authors. Study quality was classified as low, medium, or high based on the following five criteria: description of study design and setting, description of eligibility criteria, study population, description of outcomes, and description of the study participants (20). An overall score of low, medium, and high was then assigned to each study. The full criteria used to assess study quality are found in Supplementary Material 2. Disagreements were resolved through discussion or the intervention of all the authors.

### Analysis

For each included study, the overall and child prevalence of congenital myopathy per 100,000 individuals was considered the primary outcome for the meta-analysis. The overall and child prevalence of each subtype of congenital myopathy per 100,000 individuals was the secondary outcome. In addition, the proportion of mutant genes for every subtype of congenital myopathy was calculated by dividing the number of patients who had the indicated mutant genes by the total number of patients who had genetic information of certain congenital myopathies. Estimates used the population as the denominator. To be included in the meta-analysis, studies must have reported the number of cases and sample size, an estimate with confidence intervals, or the information needed to calculate the required information. As significant heterogeneity was expected, we decided to employ random effects models to complete stratified analysis along with meta-regression to investigate sources of heterogeneity. To assess significant between-study heterogeneity, the Cochrane *Q* statistic was calculated, and *I*^2^, a statistic describing the proportion of variation in point estimates due to heterogeneity of studies rather than to sampling error, was used to quantify the amount of between-study heterogeneity. Potential heterogeneity included continent, country, diagnostic criteria, and the definition of condition.

Study-specific prevalence estimates (along with their 95% CI) as well as the summary prevalence estimates were graphically represented with a forest plot: for each study, ordered by the publication year, a square was plotted whose center projection corresponded to the study-specific estimate. A diamond was used to plot the summary prevalence, the center of which represents the point estimate, whereas the extremes of the summary estimate show the 95% CI. For all tests, *p* < 0.05 was deemed significant. All statistical analyses were carried out in Review Manager 5.4 software.

## Results

### Study Selection and Characteristics

The flow chart for study selection is shown in Figure 1. Overall, the initial literature search identified 10,584 studies. Following the removal of duplicates (*n* = 6,477); 4,107 abstracts were initially screened, and only 479 (11.7%) full text articles were reviewed for further evaluation. A total of 468 studies were excluded, and the reasons for exclusion after full-text assessment are listed in Supplementary Table 1. Of these, based on a literature review, 11 (2.3%) studies containing information on the prevalence of congenital myopathy met the eligibility criteria and were therefore included for qualitative and quantitative analysis in this systematic review. The detailed characteristics of the included studies are summarized in Table 1. Eleven studies reported the prevalence of congenital myopathy (21, 22, 25, 27–29, 31, 34, 35, 37, 38). Eight studies examined the prevalence of nemaline myopathy (21, 22, 25, 28, 29, 34, 37, 38). Nine studies showed the prevalence of core myopathy (21, 22, 25, 28, 29, 31, 34, 37, 38). Five studies detected the prevalence of centronuclear myopathy (21, 28, 34, 37, 38). Seven studies calculated the prevalence of congenital fiber-type disproportion myopathy (21, 22, 25, 28, 31, 37, 38). Seven studies examined the prevalence of other congenital myopathies, such as myosin storage myopathy and other unspecified myopathies (21, 22, 25, 28, 31, 37, 38).

**Table 1:** Characteristics of the included studies on congenital myopathy prevalence.

**References**	**Country/region**	**Age (years)**	**Data source**	**Diagnostic criteria**	**Prevalence date**	**Population size**	**Number of cases**	**Prevalence per 100,000 (95% CI)**	**Overall score^d^**
Amburgey et al. (21)	United States (Michigan)	<18	Hospital/clinic chart review, administrative database	Clinical history with at least 1 additional supporting study (biopsy, genetic testing, or first-degree relative)	2010	1,211,100	46	3.80 (2.93, 4.66)	Medium
Chung et al. (22)	Southern China (Hong Kong)	<19	Hospital/clinic chart review, administrative database	European Neuromuscular Center (23), World Federation of Neurology Research Committee (24)^a^	2001.06.30	1,335,469	45	3.22 (2.43, 4.01)	Medium
Darin and Tulinius (25)	Western Sweden	<16	Mailed survey, hospital/clinic chart review, administrative databases	Muscle and Nerve (26)^b^	1995.01.01	359,676	18	5.01 (3.37, 6.64)	Medium
Hughes et al. (27)	Northern Ireland	All	Hospital/clinic chart review, administrative database, relatives.	European Neuromuscular Center (23), World Federation of Neurology Research Committee (24)^a^	1994.06.30	1,573,282	57	3.62 (2.87, 4.37)	Medium
Lefter et al. (28)	Ireland	>18	Hospital/clinic chart review, administrative database	Table e-1 at Neurology.org (28)	2013.12.31	3,439,565	33	0.96 (0.65, 1.27)	Medium
Norwood et al. (29)	Northern England	All	Hospital/clinic chart review, administrative database	European Neuromuscular Center (23), Monogenic neuromuscular disorders (30)^c^	2007.08.01	2,990,000	41	0.60 (0.33, 0.87)	Medium
Pagola-Lorz et al. (31)	Northern Spain (Navarre)	All	Hospital/clinic chart review, administrative database	Monogenic neuromuscular disorders (32), undiagnosed genetic muscle disease (33)^c^	2016	640,647	8	1.25 (0.44, 2.06)	Medium
Santos et al. (34)	Portugal	<15	NM	Details are not available	2001	1,656,602	27	1.63 (1.07, 2.19)	Low
Tangsrud and Halvorsen (35)	Southern Norway	<18	Mailed survey, hospital/clinic chart review	System proposed by Dubowitz (36)^b^	1983.01.01	573,762	3	0.52 (−0.05, 1.10)	Medium
Theadom et al. (37)	New Zealand	All	Hospital/clinic chart review, administrative database	Details are not available	2014.04.01	4,242,048	60	1.41 (1.08, 1.75)	Medium
Witting et al. (38)	Denmark	>5	Mailed survey, hospital/clinic chart review, administrative database	Highly dependent on histological findings	NM	5,400,000	82	1.52 (1.22, 1.82)	Medium

*CI, confidence interval; NM, not mentioned*.

a *Diagnosis based on characteristic histochemical abnormalities*.

b *Highly dependent on histological findings*.

c *Genetic confirmation or clinical phenotype + characteristic histological findings*.

d *Quality of study reporting assessment; details are shown in Supplementary Material 2*.

### Assessment of Study Quality

Overall, the quality of 11 studies was evaluated. In total, 10 (90.9%) studies were considered medium-quality, one (9.1%) was considered low-quality, and no study was assessed as having a high overall quality. More details about the quality of each included study are reported in Supplementary Table 2.

### Prevalence of Congenital Myopathy

Eleven studies examined the prevalence of congenital myopathy (21, 22, 25, 27–29, 31, 34, 35, 37, 38). Eight studies examined European populations, and Asian, Oceanian, and North American populations were each examined in one study separately. All the studies included service-based (including hospital records, physician surveys, and/or use of administrative databases) prevalence estimates. Four studies were based on the total population without specifying the age range, and two studies were based on the adult population specifying the age over 18 years old or 5 years old. We put these six studies into the “all” group. Five other studies were based on the child population specifying ages <15–19 years old. We categorized these five studies as the “children” group. The prevalence from the studies ranged from 0.96 to 3.62 per 100,000 in the “overall” group and 0.52–5.01 per 100,000 in the “children” group.

**Figure 2:**
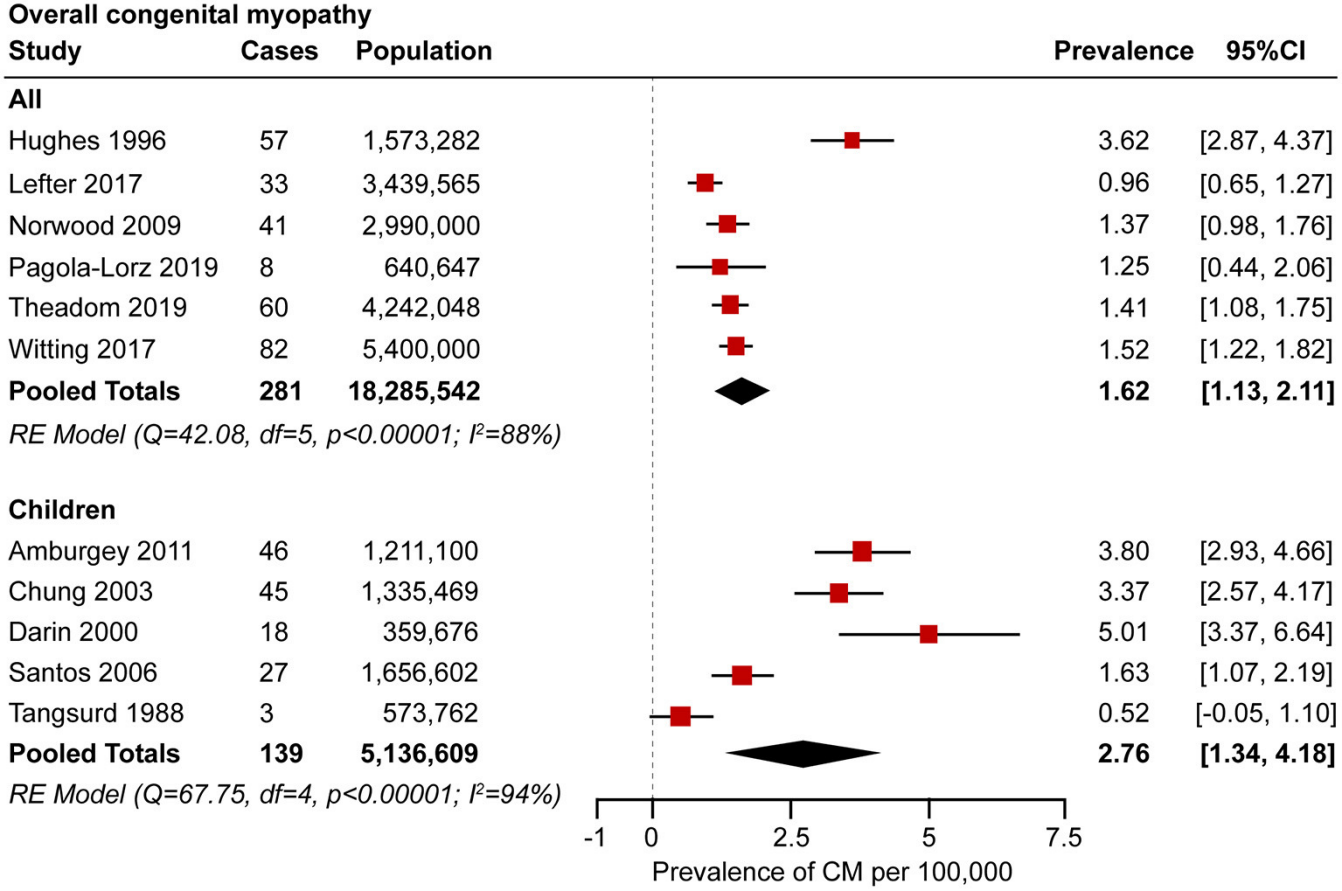
Forest plot of the estimated prevalence of congenital myopathy per 100,000 cases along with the 95% confidence interval (CI).

A meta-analysis was performed by pooling data from the 11 studies (21, 22, 25, 27–29, 31, 34, 35, 37, 38) to estimate the prevalence. Forest plots of individual studies and pooled prevalence estimates of congenital myopathy (stratified by studies including pediatric-only populations) are presented in Figure 2. Six studies were conducted in all-age populations; the overall pooled estimate on the prevalence was 1.62 (95% CI, 1.13–2.11) per 100,000. Random effects models on the meta-analyses performed showed statistically significant heterogeneity (*I*^2^ = 88%, *p* < 0.00001). Five studies were conducted in child populations; the overall prevalence was 2.76 (95% CI, 1.34–4.18) per 100,000. Random effects models on the meta-analyses performed showed statistically significant heterogeneity (*I*^2^ = 94%, *p* < 0.00001).

**Figure 3:**
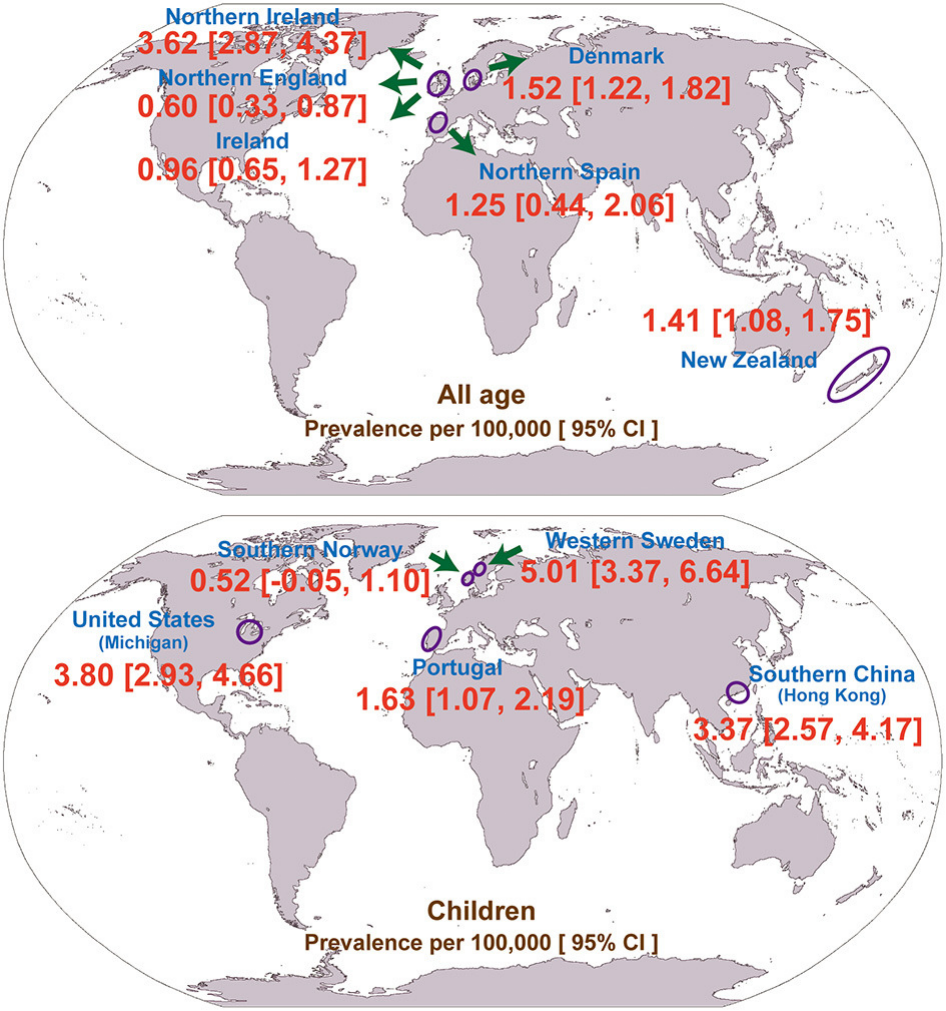
Geographical distribution of the prevalence of congenital myopathy.

Interestingly, the majority of the included studies (*N* = 8; 73%) were conducted in Europe (Table 1). In addition, we pooled the prevalence of geographically adjacent countries or districts to compare the geographical prevalence. However, no significant difference was found (chi-square test, *p* = 0.448, 0.291 for the all-age and child populations, respectively). The geographical prevalence of congenital myopathy is presented in Figure 3.

### Prevalence of Nemaline Myopathy

**Figure 4:**
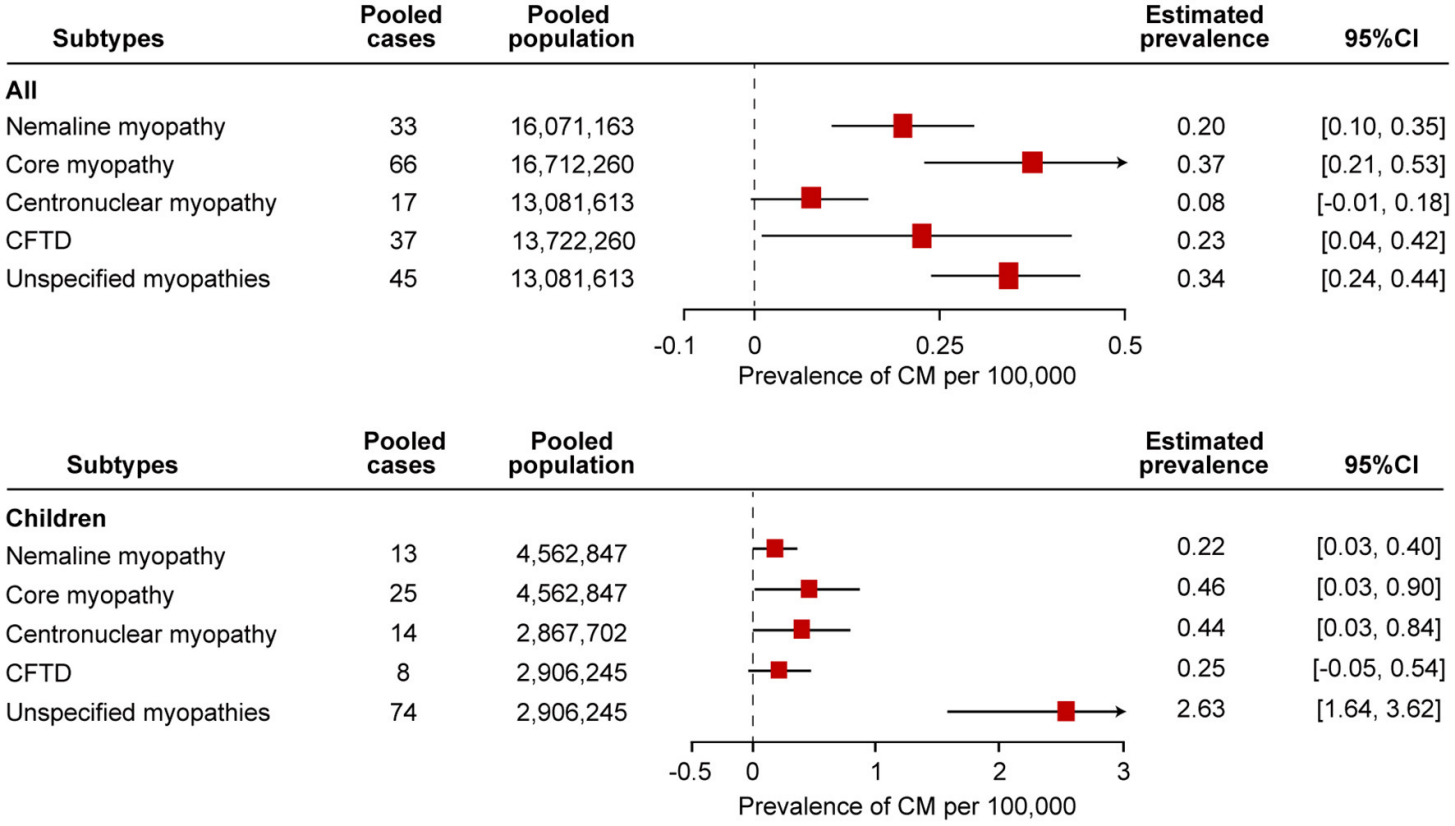
Comparison of the estimated prevalence of different subtypes of congenital myopathy per 100,000 cases along with the 95% confidence interval (CI). CFTD, congenital fiber-type disproportion myopathy.

Eight studies examined the prevalence of nemaline myopathy (21, 22, 25, 28, 29, 34, 37, 38). Four studies were based on the total population without specifying the age range or on the adult population specifying ages over 18 years old or 5 years old. Four studies were based on the child population. The prevalence of nemaline myopathy in the studies ranged from 0.14 to 0.26 per 100,000 in the “all” group and 0.08–0.56 per 100,000 in the “children” group. Forest plots of individual studies and pooled prevalence estimates of nemaline myopathy are presented in Figure 4 and Supplementary Figure 1. Four studies were conducted in all-age populations; the overall pooled estimate on the prevalence was 0.20 (95% CI, 0.10–0.35) per 100,000. Random effects models on the meta-analyses performed did not show statistically significant heterogeneity (*I*^2^ = 0%, *p* = 0.65). Another four studies were conducted in child populations; the overall prevalence was 0.22 (95% CI, 0.03–0.40) per 100,000. Random effects models on the meta-analyses performed did not show statistically significant heterogeneity (*I*^2^ = 48%, *p* = 0.13).

### Prevalence of Core Myopathy

Core myopathy is a clinically and genetically heterogeneous group of congenital myopathies, including central core myopathy, minicore myopathy, multicore myopathy, and multi-minicore myopathy. Nine studies examined the prevalence of core myopathy (21, 22, 25, 28, 29, 31, 34, 37, 38). Five studies were based on the total population without specifying the age range or on the adult population specifying the age over 18 years old or 5 years old. Another four studies were based on the child population. The prevalence from the studies ranged from 0.23 to 0.71 per 100,000 in the “all” group and 0.08–1.23 per 100,000 in the “children” group. Forest plots of individual studies and pooled prevalence estimates of core myopathy are presented in Figure 4 and Supplementary Figure 2. The overall pooled estimate on the prevalence of all-age populations was 0.37 (95% CI, 0.21–0.53) per 100,000. Random effects models on the meta-analyses performed showed statistically significant heterogeneity (*I*^2^ = 67%, *p* = 0.02). In addition, the overall prevalence of the child population was 0.46 (95% CI, 0.03–0.90) per 100,000. Random effects models on the meta-analyses performed showed statistically significant heterogeneity (*I*^2^ = 83%, *p* = 0.0005).

### Prevalence of Centronuclear Myopathy

Five studies examined the prevalence of centronuclear myopathy (21, 28, 34, 37, 38). Each study was based on the total population without specifying the age range and on the adult population specifying the age over 18 years old and 5 years old. Two studies were based on child populations. Three patients with myotubular myopathy were diagnosed in one study (34). Since myotubular myopathy is a subtype of centronuclear myopathy, we included these three patients as having centronuclear myopathy (12). The prevalence of centronuclear myopathy in the studies ranged from 0.02 to 0.28 per 100,000 in the “all” group and 0.25 to 0.66 per 100,000 in the “children” group. Forest plots of individual studies and pooled prevalence estimates of core myopathy are presented in Figure 4 and Supplementary Figure 3. The overall pooled estimate of the prevalence of centronuclear myopathy among the all-age population was 0.08 (95% CI, −0.01 to 0.18) per 100,000. Random effects models on the meta-analyses performed showed statistically significant heterogeneity (*I*^2^ = 83%, *p* = 0.003). In addition, the overall prevalence of centronuclear myopathy among the child population was 0.44 (95% CI, 0.03–0.84) per 100,000. Random effects models on the meta-analyses performed showed statistically significant heterogeneity (*I*^2^ = 67%, *p* = 0.04).

### Prevalence of Congenital Fiber-Type Disproportion Myopathy

Seven studies examined the prevalence of congenital fiber-type disproportion myopathy (21, 22, 25, 28, 31, 37, 38). Five studies were based on the total population without specifying the age range or on the adult population specifying the age over 18 years old or 5 years old. Three studies were based on the child population. The prevalence from the studies ranged from 0.06 to 0.50 per 100,000 in the “all” group and 0.08 to 0.56 per 100,000 in the “children” group. Forest plots of individual studies and pooled prevalence estimates of congenital fiber-type disproportion myopathy are presented in Figure 4 and Supplementary Figure 4. The overall pooled estimate on the prevalence of the all-age population was 0.23 (95% CI, 0.04–0.42) per 100,000. Random effects models on the meta-analyses performed showed statistically significant heterogeneity (*I*^2^ = 85%, *p* = 0.0001). In addition, the overall prevalence of the child population was 0.25 (95% CI, −0.05 to 0.54) per 100,000. Random effects models on the meta-analyses performed did not show statistically significant heterogeneity (*I*^2^ = 53%, *p* = 0.12).

### Prevalence of Unspecified Congenital Myopathies

Seven studies examined the prevalence of unspecified congenital myopathies (21, 22, 25, 28, 37, 38). Three studies were based on the total population without specifying the age range or on the adult population specifying the age over 18 years old or 5 years old. Three studies were based on the child population. The prevalence from the studies ranged from 0.26 to 0.42 per 100,000 in the “all” group and 1.82 to 3.61 per 100,000 in the “children” group. Forest plots of individual studies and pooled prevalence estimates of unspecified congenital myopathies are presented in Figure 4 and Supplementary Figure 5. The overall pooled estimate on the prevalence of the all-age population was 0.34 (95% CI, 0.24–0.44) per 100,000. Random effects models on the meta-analyses performed showed statistically significant heterogeneity (*I*^2^ = 10%, *p* < 0.00001). In addition, the overall prevalence of the child population was 2.63 (95% CI, 1.64–3.62) per 100,000. Random effects models on the meta-analyses performed showed statistically significant heterogeneity (*I*^2^ = 71%, *p* < 0.00001).

### Genetics of Congenital Myopathy

More than 30 genes were found to be associated with congenital myopathy (39–41). In the 11 enrolled studies, three studies included genetic information (28, 31, 38). A total of 123 congenital myopathies were included, 59 (48.0%) of which had genetic information. In nemaline myopathy, *ACTA1* mutations (41.2%) were the most common mutations. *RYR1* mutations account for 93.3% of core myopathy mutations. In centronuclear myopathy and congenital fiber-type disproportion myopathy, *DNM2* (46.2%) and *RYR1* (66.7%) were the most common mutations, respectively. Overall, *RYR1* (40.7%) is likely to be the most common gene causing congenital myopathy, which is consistent with other previous studies (42). Details of genetics are shown in Table 2.

**Table 2:** Genetic information of enrolled studies.

**Disease**	**Genes (n^a^; proportion^b^)**			
Nemaline myopathy	*ACTA1* (7; 41.2%)	*NEB* (6; 35.3%)	*TPM2* (3; 17.6%)	*SEPN1* (1; 5.9%)
Core myopathy	*RYR1* (14; 93.3%)	*SEPN1* (1; 6.7%)		
Centronuclear myopthy	*DNM2* (6; 46.2%)	*RYR1* (3; 23.1%)	*MTM1* (3; 23.1%)	*TTN* (1; 7.7%)
CFTD	*RYR1* (6; 66.7%)	*TPM3* (2; 22.2%)	*ACTA1* (1; 11.1%)	
Others	*MYH7* (2; 40.0%)	*RYR1* (1; 20.0%)	*SCN4A* (1; 20.0%)	*SEPN1* (1; 20.0%)

*CFTD, congenital fiber-type disproportion myopathy*.

a *n, number of patients with the indicated gene*.

b *Proportion was calculated with the denominator of the number of patients who had genetic information of certain congenital myopathies*.

## Discussion

This meta-analysis provides a broad overview of the prevalence of congenital myopathy, including an evaluation of the quality of study reporting along with testing for publication bias. To the best of our knowledge, this is the first comprehensive systematic review that evaluates the pooled prevalence of congenital myopathy. The pooled overall prevalence and child prevalence of congenital myopathy were 1.62 (95% CI, 1.13–2.11) and 2.76 (95% CI, 1.34–4.18) per 100,000, respectively.

The prevalence in children is higher than the prevalence in adults because children with congenital myopathy may not survive beyond pediatric age with low adherence to standards of care. The prevalence of congenital myopathy showed wide geographical variations (Table 1; Figure 3). In adults, the lowest reported for Northern England (0.60 per 100,000) was up to 3.62 per 100,000 for Northern Ireland. In children, the prevalence ranged from 0.52 to 5.01 per 100,000. Although all the enrolled studies were very thorough in an attempt to avoid misdiagnosis, the accuracy of these estimates could be strongly affected by different inclusion criteria and diagnostic methods, which could lead to variable prevalence estimations. Ascertainment bias, based mainly on the methods of patient collection, may also explain the regional variation in prevalence. As also discussed in the enrolled studies, the different prevalence may also be explained by rigorous family member investigation or founder effects (25).

The prevalence of myosin storage myopathy in child population was reported from only one study, which we could not conduct a meta-analysis on the prevalence of myosin storage myopathy (31). The prevalence of myosin storage myopathy in child population was 0.47 (95% CI, −0.05 to 0.99) per 100,000. Pooling the results of the different epidemiological studies, especially in orphan diseases, is particularly advantageous since this increase in the total sample size allows more precise estimates and accounts for the potential differences among the included studies. A pooled prevalence of congenital myopathy is necessary to provide a more robust estimation for other countries and regions where no prevalence was reported. Comparison of pooled estimates demonstrates that core myopathy is the most prevalent congenital myopathy in all populations, with a pooled prevalence of 0.37 (95% CI, 0.21–0.53) per 100,000. However, “unspecified congenital myopathies” is the most prevalent congenital myopathy in the pediatric population, with a pooled prevalence of 2.63 (95% CI, 1.64–3.62) per 100,000.

Due to the limitations of standard diagnosis and classification of congenital myopathy, unspecified congenital myopathies cannot be classified, which is the most prevalent congenital myopathy in child population, suggesting that a majority of unclassified or unspecified congenital myopathies still lack correct histopathologic classification. Patients in the unspecified congenital myopathies group had a suggestive family history or diagnostic testing, and a biopsy with non-specific myopathic features (21, 22, 25, 28, 37, 38). The fact that unspecified congenital myopathies are the majority subtype in the child population underscores the important point that muscle biopsy is not sufficiently diagnostic. Additional and potentially novel modalities, especially the genetic testing, are needed in many cases to establish the ultimate diagnosis (5). The prevalence of unspecified congenital myopathies in children is higher than the prevalence in adults partially due to children with unspecified congenital myopathies may hardly survive beyond pediatric age.

The majority of the studies included used real-world data sources, such as claims databases, electronic medical records, and patient/disease registries. Such data sources have a significant, and often underused, potential to study orphan diseases and to carry out accurate epidemiological evaluations (43). The main advantage of using real-world data sources is the size of the catchment population, which is often very large, on the order of millions (44). While this is an advantage in any research setting, it is particularly valuable to study orphan diseases because the incidence of these diseases is extremely low. The role of patient registers in the published literature has been acknowledged as an important real-world data source for orphan diseases for many years, although they have been underused because of barriers to data access. Registers provide a unique opportunity to follow the natural history of the disease in time (45). The main limitation of registers with regards to the prevalence of orphan diseases is that the catchment area and its population may not be clearly defined, which makes it difficult to estimate the accurate frequency of the diagnosis being made.

The limitations of this systematic review include variability in methodology, diagnostic criteria, and non-random geographic distribution among the studies. First, similar inconsistencies exist in methodology between epidemiological studies of other neurological conditions, resulting in variations in population estimates. Second, due to the published time of the included studies, the diagnostic criteria were different, leading to a selection bias. Third, muscle biopsy is the only powerful method for the diagnosis of congenital myopathy in early years and can explain their initial histopathologically oriented classification, which, although still in use, tends to be replaced by genetic terms currently in the golden era of modern genetics. The lack of genetic testing in earlier studies may lead to an imprecise estimation of the true prevalence of congenital myopathy. Fourth, despite great efforts to ensure that the search strategy was as comprehensive as possible, we did not include abstracts, gray literature, or non-English articles. Excluding non-English articles, a common practice in meta-analysis, could therefore also lead to a biased sample of primary studies. Additionally, since information on the national population-based prevalence of congenital myopathy is quite limited, non-national population-based studies were also included in our meta-analysis. These results should be interpreted in light of these limitations.

## Conclusions

This is the first meta-analysis of the minimum prevalence estimates for overall congenital myopathy and its subtypes, such as nemaline myopathy, core myopathy, centronuclear myopathy, congenital fiber-type disproportion myopathy, and other congenital myopathies, derived from studies around the world. Our pooled estimates are useful for calculating projections of expected case numbers in regions that lack accurate prevalence data, facilitating estimation of health care burden, economic impact, and clinical resource requirements. Generating the prevalence of congenital myopathy is fundamental to support public health decision-making in facilitating the estimation of health care burden, economic impact, and clinical resource requirements. The overall quality of epidemiological studies on congenital myopathy was relatively low, highlighting the need for high-quality studies in this field. Since careful attention to the combination of clinical, immunohistochemical, and molecular genetic data is required for congenital myopathy and because reliance on immunohistochemistry alone may be misleading, high-quality clinical studies with genetic classification must be conducted to calculate the prevalence of congenital myopathy.

## Data Availability Statement

The original contributions presented in the study are included in the article/Supplementary Material, further inquiries can be directed to the corresponding author/s.

## Author Contributions

KH and HY conceived the project. KH extracted data and performed the analysis with the help of F-FB and HY. KH wrote the manuscript. F-FB and HY polished the manuscript and critically revised the manuscript for valuable intellectual content. All authors approved the final manuscript.

## Funding

This study was supported by the Science and Technology Innovation Program of Hunan Province, China (Grant No. 2021RC2023, KH), the National Natural Science Foundation of China (Grant No. 82171433, F-FB) and the National Key Research and Development Program of China (Neurological Disease Cohort Study of Precision Medicine Research Project, Grant No. 2017YFC0907700, F-FB).

## Conflict of Interest

The authors declare that the research was conducted in the absence of any commercial or financial relationships that could be construed as a potential conflict of interest.

## Publisher's Note

All claims expressed in this article are solely those of the authors and do not necessarily represent those of their affiliated organizations, or those of the publisher, the editors and the reviewers. Any product that may be evaluated in this article, or claim that may be made by its manufacturer, is not guaranteed or endorsed by the publisher.
