# Corrigendum: A Systematic Review and Meta-Analysis of the Prevalence of Congenital Myopathy

**DOI:** 10.3389/fneur.2022.857959

**Published:** 2022-02-14

**Authors:** Kun Huang, Fang-Fang Bi, Huan Yang

**Affiliations:** ^1^Department of Neurology, Xiangya Hospital, Central South University, Changsha, China; ^2^Institute of Molecular Precision Medicine and Hunan Key Laboratory of Molecular Precision Medicine, Xiangya Hospital, Central South University, Changsha, China

**Keywords:** prevalence, congenital myopathy, nemaline myopathy, core myopathy, centronuclear myopathy, congenital fiber-type disproportion myopathy

In the original article, there was an error. In the Abstract there was a mistake in the statement of the results of the pooled prevalence of congenital myopathy in the all-age population. Instead of “The pooled prevalence of congenital myopathy in the all-age population was 1.50 (95% CI, 0.93–2.06) per 100,000, while the prevalence in the child population was 2.73 (95% CI, 1.34–4.12) per 100,000”, it should be “The pooled prevalence of congenital myopathy in the all-age population was 1.62 (95% CI, 1.13–2.11) per 100,000, while the prevalence in the child population was 2.76 (95% CI, 1.34–4.18) per 100,000.”

A correction has been made to *Abstract, Results, Paragraph 1*:

**Results:** A total of 11 studies were included in the systematic review and meta-analysis. Of the 11 studies included, 10 (90.9%) were considered medium-quality, one (9.1%) was considered low-quality, and no study was assessed as having a high overall quality. The pooled prevalence of congenital myopathy in the all-age population was 1.62 (95% CI, 1.13–2.11) per 100,000, while the prevalence in the child population was 2.76 (95% CI, 1.34–4.18) per 100,000. In the pediatric population, the prevalence among males was 2.92 (95% CI, −1.70 to 7.55) per 100,000, while the prevalence among females was 2.47 (95% CI, −1.67 to 6.61) per 100,000. The prevalence estimates of the all-age population per 100,000 were 0.20 (95% CI 0.10–0.35) for nemaline myopathy, 0.37 (95% CI 0.21–0.53) for core myopathy, 0.08 (95% CI −0.01 to 0.18) for centronuclear myopathy, 0.23 (95% CI 0.04–0.42) for congenital fiber-type disproportion myopathy, and 0.34 (95% CI, 0.24–0.44) for unspecified congenital myopathies. In addition, the prevalence estimates of the pediatric population per 100,000 were 0.22 (95% CI 0.03–0.40) for nemaline myopathy, 0.46 (95% CI 0.03–0.90) for core myopathy, 0.44 (95% CI 0.03–0.84) for centronuclear myopathy, 0.25 (95% CI −0.05 to 0.54) for congenital fiber-type disproportion myopathy, and 2.63 (95% CI 1.64–3.62) for unspecified congenital myopathies.

In the original article, there was a mistake in Table 1 as published. The number of cases in the reference Norwood et al. should be 41, not 18. The corrected Table 1 appears below.

**Table 1:** Characteristics of the included studies on congenital myopathy prevalence.

**References**	**Country/region**	**Age (years)**	**Data source**	**Diagnostic criteria**	**Prevalence date**	**Population size**	**Number of cases**	**Prevalence per 100,000 (95% CI)**	**Overall score^d^**
Amburgey et al. (21)	United States (Michigan)	<18	Hospital/clinic chart review, administrative database	Clinical history with at least 1 additional supporting study (biopsy, genetic testing, or first-degree relative)	2010	1,211,100	46	3.80 (2.93, 4.66)	Medium
Chung et al. (22)	Southern China (Hong Kong)	<19	Hospital/clinic chart review, administrative database	European Neuromuscular Center (23), World Federation of Neurology Research Committee (24)^a^	2001.06.30	1,335,469	45	3.22 (2.43, 4.01)	Medium
Darin and Tulinius (25)	Western Sweden	<16	Mailed survey, hospital/clinic chart review, administrative databases	Muscle and Nerve (26)^b^	1995.01.01	359,676	18	5.01 (3.37, 6.64)	Medium
Hughes et al. (27)	Northern Ireland	All	Hospital/clinic chart review, administrative database, relatives.	European Neuromuscular Center (23), World Federation of Neurology Research Committee (24)^a^	1994.06.30	1,573,282	57	3.62 (2.87, 4.37)	Medium
Lefter et al. (28)	Ireland	>18	Hospital/clinic chart review, administrative database	Table e-1 at Neurology.org (28)	2013.12.31	3,439,565	33	0.96 (0.65, 1.27)	Medium
Norwood et al. (29)	Northern England	All	Hospital/clinic chart review, administrative database	European Neuromuscular Center (23), Monogenic neuromuscular disorders (30)^c^	2007.08.01	2,990,000	41	0.60 (0.33, 0.87)	Medium
Pagola-Lorz et al. (31)	Northern Spain (Navarre)	All	Hospital/clinic chart review, administrative database	Monogenic neuromuscular disorders (32), undiagnosed genetic muscle disease (33)^c^	2016	640,647	8	1.25 (0.44, 2.06)	Medium
Santos et al. (34)	Portugal	<15	NM	Details are not available	2001	1,656,602	27	1.63 (1.07, 2.19)	Low
Tangsrud and Halvorsen (35)	Southern Norway	<18	Mailed survey, hospital/clinic chart review	System proposed by Dubowitz (36)^b^	1983.01.01	573,762	3	0.52 (−0.05, 1.10)	Medium
Theadom et al. (37)	New Zealand	All	Hospital/clinic chart review, administrative database	Details are not available	2014.04.01	4,242,048	60	1.41 (1.08, 1.75)	Medium
Witting et al. (38)	Denmark	>5	Mailed survey, hospital/clinic chart review, administrative database	Highly dependent on histological findings	NM	5,400,000	82	1.52 (1.22, 1.82)	Medium

*CI, confidence interval; NM, not mentioned*.

a *Diagnosis based on characteristic histochemical abnormalities*.

b *Highly dependent on histological findings*.

c *Genetic confirmation or clinical phenotype + characteristic histological findings*.

d *Quality of study reporting assessment; details are shown in Supplementary Material 2*.

The authors apologize for these errors and state that they do not change the scientific conclusions of the article in any way. The original article has been updated.

## Publisher's Note

All claims expressed in this article are solely those of the authors and do not necessarily represent those of their affiliated organizations, or those of the publisher, the editors and the reviewers. Any product that may be evaluated in this article, or claim that may be made by its manufacturer, is not guaranteed or endorsed by the publisher.
