# Supplementary material for: A Systematic Review and Meta-Analysis of the Prevalence of Congenital Myopathy
Source: Front Neurol. 2021 Nov 2;12:761636. doi: 10.3389/fneur.2021.761636 (PMC8592924; doi:10.3389/fneur.2021.761636)
Supplement: Supplementary file 1 [file Data_Sheet_1.docx]

**A systematic review and meta-analysis of the prevalence of congenital myopathy**

Kun Huang^1,2^, Fang-Fang Bi^1,*^, Huan Yang^1*^

^1^ *Department of Neurology, Xiangya Hospital, Central South University, Changsha, Hunan province, China*

*^2^ Institute of Molecular Precision Medicine and Hunan Key Laboratory of Molecular Precision Medicine, Xiangya Hospital, Central South University, Changsha, Hunan province, China*

***^*^ Correspondence:***

*Fang-Fang Bi (E-mail: fangfangbi@csu.edu.cn) or*

*Huan Yang (E-mail: yangh69@126.com)*

Supplementary information includes:

Supplementary Table S1-S2.

Supplementary Figure S1-S5.

**Supplementary Table S1. Excluded studies and reasons for exclusion of studies.**

| **No.** | **Title** | **First author** | **Journal** | **Year** | **Reason for exclusion** |
| --- | --- | --- | --- | --- | --- |
| 1 | Spectrum of Clinical Features in X-Linked Myotubular Myopathy Carriers: An International Questionnaire Study | Reumers SFI | Neurology | 2021 | Denominator/population is not clear |
| 2 | A Cross-Sectional Study of Nemaline Myopathy | Amburgey K | Neurology | 2021 | Denominator/population is not clear |
| 3 | The Phenotype and Genotype of Congenital Myopathies Based on a Large Pediatric Cohort | Natera-de Benito D | Pediatr Neurol | 2021 | Denominator/population is not clear |
| 4 | Short- and Long-term Outcome After Interventional VSD Closure: A Single-Center Experience in Pediatric and Adult Patients | Bergmann M | Pediatr Cardiol | 2021 | Denominator/population is not clear |
| 5 | Outcomes and Complications in Management of Congenital Myopathy Early-Onset Scoliosis | Magee L | J Pediatr Orthop | 2021 | Lack prevalence data |
| 6 | α-tropomyosin gene (TPM3) mutation in an infant with nemaline myopathy | Almobarak S | Clin Case Rep | 2021 | Lack prevalence data |
| 7 | Surgical treatment of congenital left ventricular diverticulum | Yao M | J Thorac Dis | 2021 | Lack prevalence data |
| 8 | Making sense of missense variants in TTN-related congenital myopathies | Rees M | Acta Neuropathol | 2021 | Lack prevalence data |
| 9 | The Natural History of Pediatric Trigger Thumb in the United States | Hutchinson DT | J Hand Surg Am | 2021 | Lack prevalence data |
| 10 | Responsiveness and Minimal Clinically Important Difference of the Motor Function Measure in Collagen VI-Related Dystrophies and Laminin Alpha2-Related Muscular Dystrophy | Le Goff L | Arch Phys Med Rehabil | 2021 | Lack prevalence data |
| 11 | Potential patient screening for late-onset Pompe disease in suspected sleep apnea: a rationale and study design for a Prospective Multicenter Observational Cohort Study in Japan (PSSAP-J Study) | Yamauchi M | Sleep Breath | 2021 | Lack prevalence data |
| 12 | Epilepsy in patients with advanced Fukuyama congenital muscular dystrophy | Kuwayama R | Brain Dev | 2021 | Lack prevalence data |
| 13 | Characterization of individuals with selected muscular dystrophies from the expanded pilot of the Muscular Dystrophy Surveillance, Tracking and Research Network (MD STARnet) in the United States | Wallace B | Birth Defects Res | 2021 | Lack prevalence data |
| 14 | Clinical and Genomic Evaluation of 207 Genetic Myopathies in the Indian Subcontinent | Chakravorty S | Front Neurol | 2020 | Denominator/population is not clear |
| 15 | Global FKRP Registry: observations in more than 300 patients with Limb Girdle Muscular Dystrophy R9 | Murphy LB | Ann Clin Transl Neurol | 2020 | Denominator/population is not clear |
| 16 | Natural History and Risk Stratification in Andersen-Tawil Syndrome Type 1 | Mazzanti A | J Am Coll Cardiol | 2020 | Denominator/population is not clear |
| 17 | Clinical and pathological characterization of FLNC-related myofibrillar myopathy caused by founder variant c.8129G>A in Hong Kong Chinese | Lee HH | Clin Genet | 2020 | Denominator/population is not clear |
| 18 | Orthopedic findings in arthrogryposis and congenital Zika syndrome: A case series | Serpa SC | Birth Defects Res | 2020 | Denominator/population is not clear |
| 19 | Mortality and respiratory support in X-linked myotubular myopathy: a RECENSUS retrospective analysis | Graham RJ | Arch Dis Child | 2020 | Denominator/population is not clear |
| 20 | Treating pediatric colorectal patients in low and middle income settings: Creative adaptation to the resources available | Brisighelli G | Semin Pediatr Surg | 2020 | Lack prevalence data |
| 21 | Precision Medicine in Rare Diseases | Villalón-García I | Diseases | 2020 | Lack prevalence data |
| 22 | Congenital muscular dystrophy-associated inflammatory chemokines provide axes for effective recruitment of therapeutic adult stem cell into muscles | Alexeev V | Stem Cell Res Ther | 2020 | Lack prevalence data |
| 23 | Randomized trial of lung hyperinflation therapy in children with congenital muscular dystrophy | Sawnani H | Pediatr Pulmonol | 2020 | Lack prevalence data |
| 24 | Incidence of newborn screening disorders among 56632 infants in Central Saudi Arabia. A 6-year study | Mohamed S | Saudi Med J | 2020 | Lack prevalence data |
| 25 | Genetic characteristics and follow-up of patients with fatty acid β-oxidation disorders through expanded newborn screening in a Northern Chinese population | Wang S | J Pediatr Endocrinol Metab | 2020 | Lack prevalence data |
| 26 | Clinical and genomic characteristics of LAMA2 related congenital muscular dystrophy in a patients' cohort from Qatar. A population specific founder variant | Abdel Aleem A | Neuromuscul Disord | 2020 | Lack prevalence data |
| 27 | Targeted Next-Generation Sequencing in a Large Cohort of Genetically Undiagnosed Patients with Neuromuscular Disorders in Spain | Gonzalez-Quereda L | Genes (Basel) | 2020 | Lack prevalence data |
| 28 | Epilepsy in LAMA2-related muscular dystrophy: An electro-clinico-radiological characterization | Natera-de Benito D | Epilepsia | 2020 | Lack prevalence data |
| 29 | Congenital myasthenic syndrome due to DOK7 mutation in a cohort of patients with 'unexplained' limb-girdle muscular weakness | Lorenzoni PJ | J Clin Neurosci | 2020 | Lack prevalence data |
| 30 | Patient-reported Outcomes in Arthrogryposis | Wall LB | J Pediatr Orthop | 2020 | Lack prevalence data |
| 31 | Long-Term Results of Transcatheter Closure of Large Patent Ductus Arteriosus with Severe Pulmonary Arterial Hypertension in Pediatric Patients | Shah JH | Int J Appl Basic Med Res | 2020 | Lack prevalence data |
| 32 | Causes of secondary non-alcoholic fatty liver disease in non-obese children below 10 years | Mogahed EA | Eur J Pediatr | 2020 | Lack prevalence data |
| 33 | Ophthalmological changes in hereditary spastic paraplegia and other genetic diseases with spastic paraplegia | de Freitas JL | J Neurol Sci | 2020 | Lack prevalence data |
| 34 | Identification of a novel pathogenic mutation of the MYH3 gene in a family with distal arthrogryposis type 2B | Wang WB | Mol Med Rep | 2020 | Lack prevalence data |
| 35 | Genetic factors in isolated and syndromic laryngeal cleft | Li Y | Paediatr Respir Rev | 2020 | Lack prevalence data |
| 36 | Presentation and Management Patterns of Lower Urinary Tract Symptoms in Adults Due to Rare Inherited Neuromuscular Diseases | Roth JD | Urology | 2020 | Lack prevalence data |
| 37 | Variant filtering, digenic variants, and other challenges in clinical sequencing: a lesson from fibrillinopathies | Najafi A | Clin Genet | 2020 | Lack prevalence data |
| 38 | Incidence and Clinical Features of TRPV4-Linked Axonal Neuropathies in a USA Cohort of Charcot-Marie-Tooth Disease Type 2 | Deng S | Neuromolecular Med | 2020 | Lack prevalence data |
| 39 | A clinical scoring system for congenital contractural arachnodactyly | Meerschaut I | Genet Med | 2020 | Lack prevalence data |
| 40 | Molecular and Clinical Characteristics of a National Cohort of Paediatric Duchenne Muscular Dystrophy Patients in Norway | Annexstad EJ | J Neuromuscul Dis | 2019 | Denominator/population is not clear |
| 41 | Development of an online registry for adults with arthrogryposis multiplex congenita: A protocol paper | Sawatzky B | Am J Med Genet C Semin Med Genet | 2019 | Denominator/population is not clear |
| 42 | Congenital muscular dystrophies in China | Ge L | Clin Genet | 2019 | Denominator/population is not clear |
| 43 | X-linked myotubular myopathy: A prospective international natural history study | Annoussamy M | Neurology | 2019 | Denominator/population is not clear |
| 44 | Dilated cardiomyopathy in a pediatric population: etiology and outcome predictors - a single-center experience | Ciuca C | Future Cardiol | 2019 | Denominator/population is not clear |
| 45 | Phenotype, treatment practice and outcome in the cobalamin-dependent remethylation disorders and MTHFR deficiency: Data from the E-HOD registry | Huemer M | J Inherit Metab Dis | 2019 | Denominator/population is not clear |
| 46 | Cognitive and adaptive functioning in congenital and childhood forms of myotonic dystrophy type 1: a longitudinal study | Lindeblad G | Dev Med Child Neurol | 2019 | Denominator/population is not clear |
| 47 | The myotonic dystrophy experience: a North American cross-sectional study | Hagerman KA | Muscle Nerve | 2019 | Denominator/population is not clear |
| 48 | A large multicenter study of pediatric myotonic dystrophy type 1 for evidence-based management | Lagrue E | Neurology | 2019 | Denominator/population is not clear |
| 49 | Clinical, ophthalmological, imaging and genetic features in Brazilian patients with ARSACS | Rezende Filho FM | Parkinsonism Relat Disord | 2019 | Denominator/population is not clear |
| 50 | 2018 ACC/AHA/HRS Guideline on the Evaluation and Management of Patients With Bradycardia and Cardiac Conduction Delay: A Report of the American College of Cardiology/American Heart Association Task Force on Clinical Practice Guidelines and the Heart Rhythm Society | Kusumoto FM | Circulation | 2019 | Denominator/population is not clear |
| 51 | Emergency room visits and admission rates of children with neuromuscular disorders: A 10-year experience in a medical center in Taiwan | Kao WT | Pediatr Neonatol | 2019 | Denominator/population is not clear |
| 52 | Myotonic dystrophy type 1: clinical manifestations in children and adolescents | Ho G | Arch Dis Child | 2019 | Denominator/population is not clear |
| 53 | A novel mutation of ABHD5 gene in a Chanarin Dorfman patient with unusual dermatological findings | Eskiocak AH | Lipids Health Dis | 2019 | Lack prevalence data |
| 54 | Aetiologies and temporal trends of atrioventricular block in young patients: a 20-year nationwide study | Rudbeck-Resdal J | Europace | 2019 | Lack prevalence data |
| 55 | Efficiency and Safety of Aftercare With Intrathecal Baclofen on Location | Goslinga-van der Gaag SME | Neuromodulation | 2019 | Lack prevalence data |
| 56 | Cost-effectiveness of home mechanical ventilation in children living in a developing country | Hassani SA | Anaesthesiol Intensive Ther | 2019 | Lack prevalence data |
| 57 | A Phenotypic Description of Congenital Myotonic Dystrophy using PhenoStacks | Prasad M | J Neuromuscul Dis | 2019 | Lack prevalence data |
| 58 | MYL2-associated congenital fiber-type disproportion and cardiomyopathy with variants in additional neuromuscular disease genes; the dilemma of panel testing | Marttila M | Cold Spring Harb Mol Case Stud | 2019 | Lack prevalence data |
| 59 | Pregnancy outcomes in mixed connective tissue disease: a multicentre study | Radin M | Rheumatology (Oxford) | 2019 | Lack prevalence data |
| 60 | Body composition in patients with congenital myotonic dystrophy | Ceballos-Sáenz D | Muscle Nerve | 2019 | Lack prevalence data |
| 61 | Medium- and long-term follow-up of transcatheter closure of ruptured sinus of Valsalva aneurysm in Central Europe population | Galeczka M | J Cardiol | 2019 | Lack prevalence data |
| 62 | Dysregulation of calcium metabolism in type 1 myotonic dystrophy | Hlaing PM | Intern Med J | 2019 | Lack prevalence data |
| 63 | Noncompaction cardiomyopathy is caused by a novel in-frame desmin (DES) deletion mutation within the 1A coiled-coil rod segment leading to a severe filament assembly defect | Marakhonov AV | Hum Mutat | 2019 | Lack prevalence data |
| 64 | Expanding the clinical description of autosomal recessive spastic ataxia of Charlevoix-Saguenay | Briand MM | J Neurol Sci | 2019 | Lack prevalence data |
| 65 | Congenital myopathies are mainly associated with a mild cardiac phenotype | Petri H | J Neurol | 2019 | Lack prevalence data |
| 66 | Clinical features and management of superficial fibromatoses | Kelenjian S | J Dtsch Dermatol Ges | 2019 | Lack prevalence data |
| 67 | Impact of newborn screening for very-long-chain acyl-CoA dehydrogenase deficiency on genetic, enzymatic, and clinical outcomes | Bleeker JC | J Inherit Metab Dis | 2019 | Lack prevalence data |
| 68 | Speckle-Tracking Echocardiography in Children With Duchenne Muscular Dystrophy: A Prospective Multicenter Controlled Cross-Sectional Study | Amedro P | J Am Soc Echocardiogr | 2019 | Lack prevalence data |
| 69 | Follow-up of fatty acid β-oxidation disorders in expanded newborn screening era | Janeiro P | Eur J Pediatr | 2019 | Lack prevalence data |
| 70 | ERS statement on exercise training and rehabilitation in patients with severe chronic pulmonary hypertension | Grünig E | Eur Respir J | 2019 | Lack prevalence data |
| 71 | Functional impairments, fatigue and quality of life in RYR1-related myopathies: A questionnaire study | van Ruitenbeek E | Neuromuscul Disord | 2019 | Lack prevalence data |
| 72 | Italian recommendations for diagnosis and management of congenital myasthenic syndromes | Maggi L | Neurol Sci | 2019 | Lack prevalence data |
| 73 | Cardiac Involvement in Emery-Dreifuss Muscular Dystrophy and Related Management Strategies | Wang S | Int Heart J | 2019 | Lack prevalence data |
| 74 | Dynamic transcriptomic analysis reveals suppression of PGC1α/ERRα drives perturbed myogenesis in facioscapulohumeral muscular dystrophy | Banerji CRS | Hum Mol Genet | 2019 | Lack prevalence data |
| 75 | Challenges in the treatment of fibrodysplasia ossificans progressiva | Gencer-Atalay K | Rheumatol Int | 2019 | Lack prevalence data |
| 76 | Birth Anomalies in Monozygotic and Dizygotic Twins: Results From the California Twin Registry | Yu Y | J Epidemiol | 2019 | Lack prevalence data |
| 77 | Multifocal Necrotizing Myopathy in Northern Elephant Seal ( Mirounga angustirostris) Pups, San Miguel Island, California | Spraker TR | Vet Pathol | 2019 | Lack prevalence data |
| 78 | Relationship of age to foramen of Huschke and investigation of the development of spontaneous temporomandibular joint herniation | Ertugrul S | Int J Oral Maxillofac Surg | 2019 | Lack prevalence data |
| 79 | Renal dysfunction is rare in Fukuyama congenital muscular dystrophy | Ishigaki K | Brain Dev | 2019 | Lack prevalence data |
| 80 | Longitudinal study of the activities of daily living and quality of life in Japanese patients with fibrodysplasia ossificans progressiva | Nakahara Y | Disabil Rehabil | 2019 | Lack prevalence data |
| 81 | Arthrogryposis multiplex congenita in utero: radiologic and pathologic findings | Skaria P | J Matern Fetal Neonatal Med | 2019 | Lack prevalence data |
| 82 | Ten years of screening for congenital disorders of glycosylation in Argentina: case studies and pitfalls | Asteggiano CG | Pediatr Res | 2018 | Denominator/population is not clear |
| 83 | A Roma founder BIN1 mutation causes a novel phenotype of centronuclear myopathy with rigid spine | Cabrera-Serrano M | Neurology | 2018 | Denominator/population is not clear |
| 84 | Update on muscle disease | Witherick J | J Neurol | 2018 | Denominator/population is not clear |
| 85 | Prematurity, ventricular septal defect and dysmorphisms are independent predictors of pathogenic copy number variants: a retrospective study on array-CGH results and phenotypical features of 293 children with neurodevelopmental disorders and/or multiple congenital anomalies | Maini I | Ital J Pediatr | 2018 | Denominator/population is not clear |
| 86 | A multicenter, retrospective medical record review of X-linked myotubular myopathy: The recensus study | Beggs AH | Muscle Nerve | 2018 | Denominator/population is not clear |
| 87 | Peripheral neuropathy and gastroenterologic disorders: an overview on an underrecognized association | Spagnoli C | Acta Biomed | 2018 | Lack prevalence data |
| 88 | Fontan Failure Secondary to Charcot-Marie-Tooth-Induced Phrenic Neuropathy | Abdul TY | Tex Heart Inst J | 2018 | Lack prevalence data |
| 89 | An integrated modelling methodology for estimating the prevalence of centronuclear myopathy | Vandersmissen I | Neuromuscul Disord | 2018 | Lack prevalence data |
| 90 | Impacts for Children Living with Genetic Muscle Disorders and their Parents - Findings from a Population-Based Study | Jones KM | J Neuromuscul Dis | 2018 | Lack prevalence data |
| 91 | Prenatal, Neonatal, and Early Childhood Features in Congenital Myotonic Dystrophy | Zapata-Aldana E | J Neuromuscul Dis | 2018 | Lack prevalence data |
| 92 | Development of a research platform for children with arthrogryposis multiplex congenita: study protocol for a pilot registry | Dahan-Oliel N | BMJ Open | 2018 | Lack prevalence data |
| 93 | Costochondral grafting for paediatric temporomandibular joint reconstruction: 10-year outcomes in 55 cases | Awal DH | Int J Oral Maxillofac Surg | 2018 | Lack prevalence data |
| 94 | Analysis of shared heritability in common disorders of the brain | Brainstorm Consortium | Science | 2018 | Lack prevalence data |
| 95 | Obstetrical, maternal and neonatal outcomes in pregnancies affected by muscular dystrophy | Petrangelo A | J Perinat Med | 2018 | Lack prevalence data |
| 96 | Muscle-tendon-related abnormalities detected by ultrasonography are common in symptomatic hip dysplasia | Jacobsen JS | Arch Orthop Trauma Surg | 2018 | Lack prevalence data |
| 97 | Health profile of a cohort of adults with Duchenne muscular dystrophy | Pandya S | Muscle Nerve | 2018 | Lack prevalence data |
| 98 | Risk Factors of Impaired Pulmonary Function in Arthrogryposis Multiplex Congenital Patients With Concomitant Scoliosis: A Comparison With Adolescent Idiopathic Scoliosis | Li Y | Spine (Phila Pa 1976) | 2018 | Lack prevalence data |
| 99 | Childhood-onset form of myotonic dystrophy type 1 and autism spectrum disorder: Is there comorbidity? | Angeard N | Neuromuscul Disord | 2018 | Lack prevalence data |
| 100 | Treating pediatric neuromuscular disorders: The future is now | Dowling JJ | Am J Med Genet A | 2018 | Lack prevalence data |
| 101 | Application of the International Classification of Functioning, Disability and Health system to symptoms of the Duchenne and Becker muscular dystrophies | Conway KM | Disabil Rehabil | 2018 | Lack prevalence data |
| 102 | Molecular characterization of congenital myasthenic syndromes in Spain | Natera-de Benito D | Neuromuscul Disord | 2017 | Denominator/population is not clear |
| 103 | Prevalence of myotonic dystrophy type 1 in adults in western Sweden | Lindberg C | Neuromuscul Disord | 2017 | Denominator/population is not clear |
| 104 | Muscle redox disturbances and oxidative stress as pathomechanisms and therapeutic targets in early-onset myopathies | Moulin M | Semin Cell Dev Biol | 2017 | Denominator/population is not clear |
| 105 | Early-Onset Myopathies: Clinical Findings, Prevalence of Subgroups and Diagnostic Approach in a Single Neuromuscular Referral Center in Germany | Vill K | J Neuromuscul Dis | 2017 | Lack prevalence data |
| 106 | Upholding Ethical Decision Making in Children With Life Limiting Illnesses | Glasper EA | Compr Child Adolesc Nurs | 2017 | Lack prevalence data |
| 107 | Congenital myopathies: clinical phenotypes and new diagnostic tools | Cassandrini D | Ital J Pediatr | 2017 | Lack prevalence data |
| 108 | Congenital Myasthenic Syndromes or Inherited Disorders of Neuromuscular Transmission: Recent Discoveries and Open Questions | Nicole S | J Neuromuscul Dis | 2017 | Lack prevalence data |
| 109 | A natural history study of X-linked myotubular myopathy | Amburgey K | Neurology | 2017 | Lack prevalence data |
| 110 | Survival among children with "Lethal" congenital contracture syndrome 11 caused by novel mutations in the gliomedin gene (GLDN) | Wambach JA | Hum Mutat | 2017 | Lack prevalence data |
| 111 | Correction of a splicing defect in a mouse model of congenital muscular dystrophy type 1A using a homology-directed-repair-independent mechanism | Kemaladewi DU | Nat Med | 2017 | Lack prevalence data |
| 112 | Congenital muscular dystrophies in the UK population: Clinical and molecular spectrum of a large cohort diagnosed over a 12-year period | Sframeli M | Neuromuscul Disord | 2017 | Lack prevalence data |
| 113 | Affected female carriers of MTM1 mutations display a wide spectrum of clinical and pathological involvement: delineating diagnostic clues | Biancalana V | Acta Neuropathol | 2017 | Lack prevalence data |
| 114 | Truncating mutations on myofibrillar myopathies causing genes as prevalent molecular explanations on patients with dilated cardiomyopathy | Janin A | Clin Genet | 2017 | Lack prevalence data |
| 115 | Long-term functional and mobility outcomes for individuals with arthrogryposis multiplex congenita | Nouraei H | Am J Med Genet A | 2017 | Lack prevalence data |
| 116 | Establishing prevalence in rare neuromuscular diseases: A lesson from congenital myopathies | Bamaga AK | Neurol Genet | 2017 | Lack prevalence data |
| 117 | Clinical course and cardiovascular outcomes in patients with the long-chain 3-hydroxyacyl-coenzyme A dehydrogenase deficiency | Kwiatkowska J | Cardiol J | 2017 | Lack prevalence data |
| 118 | Comprehensive target capture/next-generation sequencing as a second-tier diagnostic approach for congenital muscular dystrophy in Taiwan | Liang WC | PLoS One | 2017 | Lack prevalence data |
| 119 | Autism spectrum disorder and other neurobehavioural comorbidities in rare disorders of the Ras/MAPK pathway | Garg S | Dev Med Child Neurol | 2017 | Lack prevalence data |
| 120 | Anesthetic Outcomes of Children With Arthrogryposis Syndromes: No Evidence of Hyperthermia | Gleich SJ | Anesth Analg | 2017 | Lack prevalence data |
| 121 | Comparison of sitting and supine forced vital capacity in collagen VI-related dystrophy and laminin α2-related dystrophy | Meilleur KG | Pediatr Pulmonol | 2017 | Lack prevalence data |
| 122 | Dental and Temporomandibular Joint Pathology of the California Mountain Lion (Puma concolor couguar) | Aghashani A | J Comp Pathol | 2017 | Lack prevalence data |
| 123 | Cardiac Abnormalities in Congenital and Childhood Myotonic Muscular Dystrophy Type 1 | Sharma A | Neuropediatrics | 2017 | Lack prevalence data |
| 124 | Association of Transition Readiness to Intentional Self-Regulation and Hopeful Future Expectations in Youth With Illness | Hart LC | Acad Pediatr | 2017 | Lack prevalence data |
| 125 | Childhood macrophagic myofasciitis: A series from the Indian subcontinent | Kakkar A | Muscle Nerve | 2017 | Lack prevalence data |
| 126 | Physical function and mobility in children with congenital myotonic dystrophy | Pucillo EM | Muscle Nerve | 2017 | Lack prevalence data |
| 127 | Muscular dystrophies and myopathies: the spectrum of mutated genes in the Czech Republic | Stehlíková K | Clin Genet | 2017 | Lack prevalence data |
| 128 | Disease burden and functional outcomes in congenital myotonic dystrophy: A cross-sectional study | Johnson NE | Neurology | 2016 | Denominator/population is not clear |
| 129 | Muscular dystrophy in a dish: engineered human skeletal muscle mimetics for disease modeling and drug discovery | Smith AST | Drug Discov Today | 2016 | Denominator/population is not clear |
| 130 | Parent-reported multi-national study of the impact of congenital and childhood onset myotonic dystrophy | Johnson NE | Dev Med Child Neurol | 2016 | Denominator/population is not clear |
| 131 | United States Physical Therapists' Knowledge About Joint Hypermobility Syndrome Compared with Fibromyalgia and Rheumatoid Arthritis | Russek LN | Physiother Res Int | 2016 | Denominator/population is not clear |
| 132 | Arthrogryposis multiplex congenital - multidisciplinary care - including own experience | Binkiewicz-Glińska A | Dev Period Med | 2016 | Lack prevalence data |
| 133 | MYH7-related myopathies: clinical, histopathological and imaging findings in a cohort of Italian patients | Fiorillo C | Orphanet J Rare Dis | 2016 | Lack prevalence data |
| 134 | Target resequencing of neuromuscular disease-related genes using next-generation sequencing for patients with undiagnosed early-onset neuromuscular disorders | Kitamura Y | J Hum Genet | 2016 | Lack prevalence data |
| 135 | Coexistence of central nucleus, cores, and rods: Diagnostic relevance | Dhinakaran S | Ann Indian Acad Neurol | 2016 | Lack prevalence data |
| 136 | The Effect of the Presence of Fibromyalgia on Common Clinical Disease Activity Indices in Patients with Psoriatic Arthritis: A Cross-sectional Study | Brikman S | J Rheumatol | 2016 | Lack prevalence data |
| 137 | MTHFR: Addressing Genetic Counseling Dilemmas Using Evidence-Based Literature | Levin BL | J Genet Couns | 2016 | Lack prevalence data |
| 138 | Magnetic Resonance Imaging of Temporomandibular Joints of Children | Moe JS | J Oral Maxillofac Surg | 2016 | Lack prevalence data |
| 139 | A Study of a Cohort of X-Linked Myotubular Myopathy at the Clinical, Histologic, and Genetic Levels | Abath Neto O | Pediatr Neurol | 2016 | Lack prevalence data |
| 140 | DNM2 mutations in Chinese Han patients with centronuclear myopathy | Lin P | Neurol Sci | 2016 | Lack prevalence data |
| 141 | SEPN1-related myopathy in three patients: novel mutations and diagnostic clues | Ardissone A | Eur J Pediatr | 2016 | Lack prevalence data |
| 142 | Establishing a Role for Polysomnography in Hospitalized Children | Tkachenko N | Pediatr Neurol | 2016 | Lack prevalence data |
| 143 | Expanding the MYBPC1 phenotypic spectrum: a novel homozygous mutation causes arthrogryposis multiplex congenita | Ekhilevitch N | Clin Genet | 2016 | Lack prevalence data |
| 144 | Isotretinoin treatment of autosomal recessive congenital ichthyosis complicated by coexisting dysferlinopathy | Mashiah J | Clin Exp Dermatol | 2016 | Lack prevalence data |
| 145 | Characterization of a group unrelated patients with arthrogryposis multiplex congenita | Valdés-Flores M | J Pediatr (Rio J) | 2016 | Lack prevalence data |
| 146 | Expanding the spectrum of congenital anomalies of the diencephalic-mesencephalic junction | Severino M | Neuroradiology | 2016 | Lack prevalence data |
| 147 | Frame shift mutations of the ZMPSTE24 gene in two siblings with restrictive dermopathy | Matulevičienė A | Clin Dysmorphol | 2016 | Lack prevalence data |
| 148 | Suspected myofibrillar myopathy in Arabian horses with a history of exertional rhabdomyolysis | Valberg SJ | Equine Vet J | 2016 | Lack prevalence data |
| 149 | Prevalence and phenotypes of congenital myopathy due to α-actin 1 gene mutations | Witting N | Muscle Nerve | 2016 | Lack prevalence data |
| 150 | Frequency and Phenotype of Myotubular Myopathy Amongst Danish Patients with Congenital Myopathy Older than 5 Years | Werlauff U | J Neuromuscul Dis | 2015 | Denominator/population is not clear |
| 151 | Centronuclear myopathies: genotype-phenotype correlation and frequency of defined genetic forms in an Italian cohort | Fattori F | J Neurol | 2015 | Denominator/population is not clear |
| 152 | The Epidemiology of Neuromuscular Disorders: A Comprehensive Overview of the Literature | Deenen JC | J Neuromuscul Dis | 2015 | Denominator/population is not clear |
| 153 | Novel mutations in LMNA A/C gene and associated phenotypes | Petillo R | Acta Myol | 2015 | Lack prevalence data |
| 154 | Effect of distraction osteogenesis in patient with tibial shortening after initial union of Congenital Pseudarthrosis of the Tibia (CPT): a preliminary study | Zhu GH | BMC Musculoskelet Disord | 2015 | Lack prevalence data |
| 155 | Management of idiopathic pediatric syringohydromyelia | Rodriguez A | J Neurosurg Pediatr | 2015 | Lack prevalence data |
| 156 | The Use of an Hourglass Dorsal Advancement Flap Without Skin Graft for Congenital Syndactyly | Ni F | J Hand Surg Am | 2015 | Lack prevalence data |
| 157 | Complications of vitamin D deficiency from the foetus to the infant: One cause, one prevention, but who's responsibility? | Högler W | Best Pract Res Clin Endocrinol Metab | 2015 | Lack prevalence data |
| 158 | A comprehensive genetic diagnosis of Chinese muscular dystrophy and congenital myopathy patients by targeted next-generation sequencing | Dai Y | Neuromuscul Disord | 2015 | Lack prevalence data |
| 159 | Under-recognition of Low Blood Pressure Readings in Patients with Duchenne Muscular Dystrophy | Masood SA | Pediatr Cardiol | 2015 | Lack prevalence data |
| 160 | Clinical, morphological, biochemical, imaging and outcome parameters in 21 individuals with mitochondrial maintenance defect related to FBXL4 mutations | Huemer M | J Inherit Metab Dis | 2015 | Lack prevalence data |
| 161 | Summary of the 2nd International Symposium on Arthrogryposis, St. Petersburg, Russia, September 17-19, 2014 | Hall JG | Am J Med Genet A | 2015 | Lack prevalence data |
| 162 | Degenerative Cervical Myelopathy: Epidemiology, Genetics, and Pathogenesis | Nouri A | Spine (Phila Pa 1976) | 2015 | Lack prevalence data |
| 163 | Anticipation in myotonic dystrophy type 1 parents with small CTG expansions | Pratte A | Am J Med Genet A | 2015 | Lack prevalence data |
| 164 | LAMA2-related myopathy: Frequency among congenital and limb-girdle muscular dystrophies | Løkken N | Muscle Nerve | 2015 | Lack prevalence data |
| 165 | Congenital heart disease in adolescents with gluteal muscle contracture | You T | Medicine (Baltimore) | 2015 | Lack prevalence data |
| 166 | Prevalence of congenital muscular dystrophy in Italy: a population study | Graziano A | Neurology | 2015 | Lack prevalence data |
| 167 | Utility of next generation sequencing in genetic diagnosis of early onset neuromuscular disorders | Chae JH | J Med Genet | 2015 | Lack prevalence data |
| 168 | Advances in the Care of Adults With Congenital Heart Disease | Nasr VG | Semin Cardiothorac Vasc Anesth | 2015 | Lack prevalence data |
| 169 | Cystic fibrosis newborn screening: a model for neuromuscular disease screening? | Scully MA | Ann Neurol | 2015 | Lack prevalence data |
| 170 | A shared founder mutation underlies lethal restrictive dermopathy in the Austronesian aboriginal Atayal tribe of Taiwan | Hou JW | J Formos Med Assoc | 2015 | Lack prevalence data |
| 171 | Parental age effects, but no evidence for an intrauterine effect in the transmission of myotonic dystrophy type 1 | Morales F | Eur J Hum Genet | 2015 | Lack prevalence data |
| 172 | Diseases of captive yellow seahorse Hippocampus kuda Bleeker, pot-bellied seahorse Hippocampus abdominalis Lesson and weedy seadragon Phyllopteryx taeniolatus (Lacépède) | LePage V | J Fish Dis | 2015 | Lack prevalence data |
| 173 | The importance of genetic susceptibility in Dupuytren's disease. | Becker K | Clin Genet | 2015 | Lack prevalence data |
| 174 | Gene therapy for inherited muscle diseases: where genetics meets rehabilitation medicine | Braun R | Am J Phys Med Rehabil | 2014 | Denominator/population is not clear |
| 175 | Novel RYR1 missense mutations in six Chinese patients with central core disease | Gu M | Neurosci Lett | 2014 | Denominator/population is not clear |
| 176 | Fatigue in patients with spinal muscular atrophy type II and congenital myopathies: evaluation of the fatigue severity scale | Werlauff U | Qual Life Res | 2014 | Denominator/population is not clear |
| 177 | Prevalence and effect of oxytetracycline on congenital fetlock knuckling in neonatal dairy calves | Fazili MR | Onderstepoort J Vet Res | 2014 | Lack prevalence data |
| 178 | Cellular and morphological aspects of fibrodysplasia ossificans progressiva. Lessons of formation, repair, and bone bioengineering | Martelli A | Organogenesis | 2014 | Lack prevalence data |
| 179 | Cardiac manifestations of primary mitochondrial disorders | Finsterer J | Int J Cardiol | 2014 | Lack prevalence data |
| 180 | Spontaneous coronary artery dissection: novel diagnostic insights from large series of patients | Alfonso F | Circ Cardiovasc Interv | 2014 | Lack prevalence data |
| 181 | Perosomus elumbis in Danish Holstein cattle | Agerholm JS | BMC Vet Res | 2014 | Lack prevalence data |
| 182 | Genetic cerebellar ataxias | Storey E | Semin Neurol | 2014 | Lack prevalence data |
| 183 | Oligohydramnios sequence revisited in relationship to arthrogryposis, with distinctive skin changes | Hall JG | Am J Med Genet A | 2014 | Lack prevalence data |
| 184 | Bowel-associated dermatosis-arthritis syndrome in an adolescent with short bowel syndrome | Pereira E | J Clin Rheumatol | 2014 | Lack prevalence data |
| 185 | Associated morbidity of pediatric ptosis - a large, community based case-control study | Nemet AY | Graefes Arch Clin Exp Ophthalmol | 2014 | Lack prevalence data |
| 186 | Deoxynucleoside stress exacerbates the phenotype of a mouse model of mitochondrial neurogastrointestinal encephalopathy | Garcia-Diaz B | Brain | 2014 | Lack prevalence data |
| 187 | A simple surgical technique for closure of apical muscular ventricular septal defect | Mishra A | J Thorac Cardiovasc Surg | 2014 | Lack prevalence data |
| 188 | Frequency and phenotype of patients carrying TPM2 and TPM3 gene mutations in a cohort of 94 patients with congenital myopathy | Citirak G | Neuromuscul Disord | 2014 | Lack prevalence data |
| 189 | Amyoplasia revisited | Hall JG | Am J Med Genet A | 2014 | Lack prevalence data |
| 190 | Gene therapy prolongs survival and restores function in murine and canine models of myotubular myopathy | Childers MK | Sci Transl Med | 2014 | Lack prevalence data |
| 191 | How low an LDL-C should we go with statin therapy? | Kostis WJ | Curr Atheroscler Rep | 2014 | Lack prevalence data |
| 192 | The efficacy of rib-based distraction with VEPTR in the treatment of early-onset scoliosis in patients with arthrogryposis | Astur N | J Pediatr Orthop | 2014 | Lack prevalence data |
| 193 | Molecular diagnosis of mitochondrial respiratory chain disorders in Japan: focusing on mitochondrial DNA depletion syndrome | Yamazaki T | Pediatr Int | 2014 | Lack prevalence data |
| 194 | Megaesophagus in Friesian horses associated with muscular hypertrophy of the caudal esophagus | Komine M | Vet Pathol | 2014 | Lack prevalence data |
| 195 | Carrier rates of four single-gene disorders in Croatian Bayash Roma | Barešić A | Genet Test Mol Biomarkers | 2014 | Lack prevalence data |
| 196 | New ZMPSTE24 (FACE1) mutations in patients affected with restrictive dermopathy or related progeroid syndromes and mutation update | Navarro CL | Eur J Hum Genet | 2014 | Lack prevalence data |
| 197 | Isolated ventricular septal defects in the era of advanced fetal echocardiography: risk of chromosomal anomalies and spontaneous closure rate from diagnosis to age of 1 year | Gómez O | Ultrasound Obstet Gynecol | 2014 | Lack prevalence data |
| 198 | Congenital posterior pole cataract and adult onset dilating cardiomyopathy: expanding the phenotype of αB-crystallinopathies | van der Smagt JJ | Clin Genet | 2014 | Lack prevalence data |
| 199 | Epidemiology of muscular dystrophies in the Mediterranean area | Topaloglu H | Acta Myol | 2013 | Denominator/population is not clear |
| 200 | Limb-girdle muscular dystrophy type 2I is not rare in Taiwan | Liang WC | Neuromuscul Disord | 2013 | Denominator/population is not clear |
| 201 | New insights about the incidence, multisystem manifestations, and care of patients with congenital myotonic dystrophy | Hilbert JE | J Pediatr | 2013 | Denominator/population is not clear |
| 202 | Medical complication in adults with spina bifida | Werhagen L | Clin Neurol Neurosurg | 2013 | Denominator/population is not clear |
| 203 | Natural history of pulmonary function in collagen VI-related myopathies | Foley AR | Brain | 2013 | Lack prevalence data |
| 204 | Pathomorphologic findings in left ventricular hypertrabeculation/noncompaction of adults in relation to neuromuscular disorders | Gerger D | Int J Cardiol | 2013 | Lack prevalence data |
| 205 | Highly efficient in vivo delivery of PMO into regenerating myotubes and rescue in laminin-α2 chain-null congenital muscular dystrophy mice | Aoki Y | Hum Mol Genet | 2013 | Lack prevalence data |
| 206 | Congenital heart disease in Nigerian children: a multicenter echocardiographic study | Sadoh WE | World J Pediatr Congenit Heart Surg | 2013 | Lack prevalence data |
| 207 | Congenital cerebral palsy and prenatal exposure to self-reported maternal infections, fever, or smoking | Streja E | Am J Obstet Gynecol | 2013 | Lack prevalence data |
| 208 | Mutations in KLHL40 are a frequent cause of severe autosomal-recessive nemaline myopathy | Ravenscroft G | Am J Hum Genet | 2013 | Lack prevalence data |
| 209 | Rapidly progressive scoliosis and respiratory deterioration in Ullrich congenital muscular dystrophy | Yonekawa T | J Neurol Neurosurg Psychiatry | 2013 | Lack prevalence data |
| 210 | Congenital myotonic dystrophy: Canadian population-based surveillance study | Campbell C | J Pediatr | 2013 | Lack prevalence data |
| 211 | Myotonic dystrophies type 1 and 2: anesthetic care | Veyckemans F | Paediatr Anaesth | 2013 | Lack prevalence data |
| 212 | Schmallenberg virus pathogenesis, tropism and interaction with the innate immune system of the host | Varela M | PLoS Pathog | 2013 | Lack prevalence data |
| 213 | Occurrence of tendon pathologies in metabolic disorders | Abate M | Rheumatology (Oxford) | 2013 | Lack prevalence data |
| 214 | Diversity of ARSACS mutations in French-Canadians | Thiffault I | Can J Neurol Sci | 2013 | Lack prevalence data |
| 215 | Scapuloperoneal muscular dystrophy phenotype due to TRIM32-sarcotubular myopathy in South Dakota Hutterite | Liewluck T | Neuromuscul Disord | 2013 | Lack prevalence data |
| 216 | Congenital myopathy with focal loss of cross-striations revisited | Voermans NC | Neuromuscul Disord | 2013 | Lack prevalence data |
| 217 | Targeted array comparative genomic hybridization--a new diagnostic tool for the detection of large copy number variations in nemaline myopathy-causing genes | Kiiski K | Neuromuscul Disord | 2013 | Lack prevalence data |
| 218 | DOK7 limb-girdle myasthenic syndrome mimicking congenital muscular dystrophy | Mahjneh I | Neuromuscul Disord | 2013 | Lack prevalence data |
| 219 | Frequency of bovine congenital pseudomyotonia carriers in selected Italian Chianina sires | Murgiano L | Vet J | 2013 | Lack prevalence data |
| 220 | Genetic abnormalities in fibrodysplasia ossificans progressiva | Miao J | Genes Genet Syst | 2012 | Denominator/population is not clear |
| 221 | Respiratory and cardiac function in congenital muscular dystrophies with alpha dystroglycan deficiency | Pane M | Neuromuscul Disord | 2012 | Denominator/population is not clear |
| 222 | Fibrodysplasia ossificans progressiva in Spain: epidemiological, clinical, and genetic aspects | Morales-Piga A | Bone | 2012 | Lack prevalence data |
| 223 | The spectrum of myotonic and myopathic disorders in a pediatric electromyography laboratory over 12 years | Shah DU | Pediatr Neurol | 2012 | Lack prevalence data |
| 224 | Identification of congenital muscular dystonia 2 associated with an inherited GlyT2 defect in Belgian Blue cattle from the United Kingdom | Gill JL | Anim Genet | 2012 | Lack prevalence data |
| 225 | Pregnancy course and outcome in women with hereditary neuromuscular disorders: comparison of obstetric risks in 178 patients | Awater C | Eur J Obstet Gynecol Reprod Biol | 2012 | Lack prevalence data |
| 226 | Sniff nasal inspiratory pressure and sleep disordered breathing in childhood neuromuscular disorders | Anderson VB | Neuromuscul Disord | 2012 | Lack prevalence data |
| 227 | Efficacy and tolerance of gastrostomy feeding in Japanese muscular dystrophy patients | Mizuno T | Brain Dev | 2012 | Lack prevalence data |
| 228 | Severe muscle damage following viral infection in patients with Fukuyama congenital muscular dystrophy | Murakami T | Brain Dev | 2012 | Lack prevalence data |
| 229 | Variability in the clinical management of fatty acid oxidation disorders: results of a survey of Canadian metabolic physicians | Potter BK | J Inherit Metab Dis | 2012 | Lack prevalence data |
| 230 | Muscle-specific expression of insulin-like growth factor 1 improves outcome in Lama2Dy-w mice, a model for congenital muscular dystrophy type 1A | Kumar A | Hum Mol Genet | 2011 | Denominator/population is not clear |
| 231 | Fibrodysplasia ossificans progressiva: clinical and genetic aspects | Pignolo RJ | Orphanet J Rare Dis | 2011 | Lack prevalence data |
| 232 | Arthrogryposis multiplexa congenita: an epidemiologic study of nearly 9 million births in 24 EUROCAT registers | Hoff JM | Eur J Obstet Gynecol Reprod Biol | 2011 | Lack prevalence data |
| 233 | Clinical and genetic spectrum of mitochondrial neurogastrointestinal encephalomyopathy | Garone C | Brain | 2011 | Lack prevalence data |
| 234 | Fibromyalgia in fragile X mental retardation 1 gene premutation carriers | Leehey MA | Rheumatology (Oxford) | 2011 | Lack prevalence data |
| 235 | Mitochondrial neurogastrointestinal encephalopathy in an Indian family with possible manifesting carriers of heterozygous TYMP mutation | Nalini A | J Neurol Sci | 2011 | Lack prevalence data |
| 236 | Excess comorbidities associated with malignant hyperthermia diagnosis in pediatric hospital discharge records | Li G | Paediatr Anaesth | 2011 | Lack prevalence data |
| 237 | A congenital muscular dystrophy with mitochondrial structural abnormalities caused by defective de novo phosphatidylcholine biosynthesis | Mitsuhashi S | Am J Hum Genet | 2011 | Lack prevalence data |
| 238 | Autosomal recessive spastic ataxia of Charlevoix-Saguenay (ARSACS): typical clinical and neuroimaging features in a Brazilian family | Pedroso JL | Arq Neuropsiquiatr | 2011 | Lack prevalence data |
| 239 | Endplate structure and parameters of neuromuscular transmission in sporadic centronuclear myopathy associated with myasthenia | Liewluck T | Neuromuscul Disord | 2011 | Lack prevalence data |
| 240 | Frequency of the allelic variant of the PTPLA gene responsible for centronuclear myopathy in Labrador Retriever dogs as assessed in Italy | Gentilini F | J Vet Diagn Invest | 2011 | Lack prevalence data |
| 241 | Distribution of motor types in cerebral palsy: how do registry data compare? | Reid SM | Dev Med Child Neurol | 2011 | Lack prevalence data |
| 242 | Fukutin mutations in non-Japanese patients with congenital muscular dystrophy: less severe mutations predominate in patients with a non-Walker-Warburg phenotype | Yis U | Neuromuscul Disord | 2011 | Lack prevalence data |
| 243 | Clinical and laboratory aspects of Ro/SSA-52 autoantibodies | Defendenti C | Autoimmun Rev | 2011 | Lack prevalence data |
| 244 | Congenital muscular dystrophies with cognitive impairment. A population study | Messina S | Neurology | 2010 | Denominator/population is not clear |
| 245 | Prevalence of multiple congenital contractures including arthrogryposis multiplex congenita in Alberta, Canada, and a strategy for classification and coding | Lowry RB | Birth Defects Res A Clin Mol Teratol | 2010 | Lack prevalence data |
| 246 | Late deformities following the transfer of the flexor carpi ulnaris to the extensor carpi radialis brevis in children with cerebral palsy | Patterson JM | J Hand Surg Am | 2010 | Lack prevalence data |
| 247 | Osteochondral diseases and fibrodysplasia ossificans progressiva | Morales-Piga A | Adv Exp Med Biol | 2010 | Lack prevalence data |
| 248 | Hereditary channelopathies in neurology | Jurkat-Rott K | Adv Exp Med Biol | 2010 | Lack prevalence data |
| 249 | Genetic screening in the Persian Jewish community: A pilot study | Kaback M | Genet Med | 2010 | Lack prevalence data |
| 250 | Causes of mortality in northern fur seals (Callorhinus ursinus), St. Paul Island, Pribilof Islands, Alaska, 1986-2006 | Spraker TR | J Wildl Dis | 2010 | Lack prevalence data |
| 251 | Cardiac findings in congenital muscular dystrophies | Finsterer J | Pediatrics | 2010 | Lack prevalence data |
| 252 | Moebius sequence and autism spectrum disorders--less frequently associated than formerly thought | Briegel W | Res Dev Disabil | 2010 | Lack prevalence data |
| 253 | Fukutin mutations in congenital muscular dystrophies with defective glycosylation of dystroglycan in Korea | Lim BC | Neuromuscul Disord | 2010 | Lack prevalence data |
| 254 | Current concepts in pediatric temporomandibular joint disorders: Part 1. Etiology, epidemiology, and classification | Allori AC | Plast Reconstr Surg | 2010 | Lack prevalence data |
| 255 | Mitochondrial neurogastrointestinal encephalomyopathy associated with progressive hearing loss | Hiraki N | J Laryngol Otol | 2010 | Lack prevalence data |
| 256 | Limb-girdle and congenital muscular dystrophies: current diagnostics, management, and emerging technologies | Rocha CT | Curr Neurol Neurosci Rep | 2010 | Lack prevalence data |
| 257 | Neuromuscular manifestations in hereditary haemochromatosis | Wouthuis SF | J Neurol | 2010 | Lack prevalence data |
| 258 | Visual function in congenital and childhood myotonic dystrophy type 1 | Ekström AB | Ophthalmology | 2010 | Lack prevalence data |
| 259 | Adolescent health in medieval Serbia: signs of infectious diseases and risk of trauma | Djurić M | Homo | 2010 | Lack prevalence data |
| 260 | Genes causing inherited forms of cardiomyopathies. A current compendium | Paul M | Herz | 2009 | Denominator/population is not clear |
| 261 | Congenital muscular dystrophies with defective glycosylation of dystroglycan: a population study | Mercuri E | Neurology | 2009 | Denominator/population is not clear |
| 262 | Merosin-deficient congenital muscular dystrophy in Korea | Chae JH | Brain Dev | 2009 | Denominator/population is not clear |
| 263 | Cardiovascular dysfunction with low cardiac output due to a small heart in patients with chronic fatigue syndrome | Miwa K | Intern Med | 2009 | Lack prevalence data |
| 264 | What we do not know about pregnancy in hereditary neuromuscular disorders | Argov Z | Neuromuscul Disord | 2009 | Lack prevalence data |
| 265 | Mutations of the noggin (NOG) and of the activin A type I receptor (ACVR1) genes in a series of twenty-seven French fibrodysplasia ossificans progressiva (FOP) patients | Lucotte G | Genet Couns | 2009 | Lack prevalence data |
| 266 | Fukutin gene mutations in an Italian patient with early onset muscular dystrophy but no central nervous system involvement | Saredi S | Muscle Nerve | 2009 | Lack prevalence data |
| 267 | Further evidence of Fukutin mutations as a cause of childhood onset limb-girdle muscular dystrophy without mental retardation | Puckett RL | Neuromuscul Disord | 2009 | Lack prevalence data |
| 268 | Reflections on the brainstem dysfunction in neurologically disabled children | Saito Y | Brain Dev | 2009 | Lack prevalence data |
| 269 | Four Caucasian patients with mutations in the fukutin gene and variable clinical phenotype | Vuillaumier-Barrot S | Neuromuscul Disord | 2009 | Lack prevalence data |
| 270 | Mutational analysis of fukutin gene in dilated cardiomyopathy and hypertrophic cardiomyopathy | Arimura T | Circ J | 2009 | Lack prevalence data |
| 271 | Phenotypic spectrum of Fukutinopathy: most severe phenotype of Fukutinopathy | Yoshioka M | Brain Dev | 2009 | Lack prevalence data |
| 272 | Autosomal recessive tubular aggregate myopathy in an Indian family | Pandit L | Eur J Paediatr Neurol | 2009 | Lack prevalence data |
| 273 | Ethnically diverse causes of Walker-Warburg syndrome (WWS): FCMD mutations are a more common cause of WWS outside of the Middle East | Manzini MC | Hum Mutat | 2008 | Lack prevalence data |
| 274 | Muscle protein alterations in LGMD2I patients with different mutations in the Fukutin-related protein gene | Yamamoto LU | J Histochem Cytochem | 2008 | Lack prevalence data |
| 275 | Prevalence of undiagnosed congenital cardiac defects in older children | Gupta-Malhotra M | Cardiol Young | 2008 | Lack prevalence data |
| 276 | Native American myopathy: congenital myopathy with cleft palate, skeletal anomalies, and susceptibility to malignant hyperthermia | Stamm DS | Am J Med Genet A | 2008 | Lack prevalence data |
| 277 | Prenatal course of isolated muscular ventricular septal defects diagnosed only by color Doppler sonography: single-institution experience | Bahtiyar MO | J Ultrasound Med | 2008 | Lack prevalence data |
| 278 | Primary myopathies and the heart | Finsterer J | Scand Cardiovasc J | 2008 | Lack prevalence data |
| 279 | Effect of neurologic complications on outcome after heart transplant | van de Beek D | Arch Neurol | 2008 | Lack prevalence data |
| 280 | Proximal tibial osteochondromas in patients with fibrodysplasia ossificans progressiva | Deirmengian GK | J Bone Joint Surg Am | 2008 | Lack prevalence data |
| 281 | Autism spectrum conditions in myotonic dystrophy type 1: a study on 57 individuals with congenital and childhood forms | Ekström AB | Am J Med Genet B Neuropsychiatr Genet | 2008 | Lack prevalence data |
| 282 | Dystrophin deficiency in Drosophila reduces lifespan and causes a dilated cardiomyopathy phenotype | Taghli-Lamallem O | Aging Cell | 2008 | Lack prevalence data |
| 283 | Musculoskeletal complications of neuromuscular disease in children | Driscoll SW | Phys Med Rehabil Clin N Am | 2008 | Lack prevalence data |
| 284 | Diagnosis and etiology of congenital muscular dystrophy | Peat RA | Neurology | 2008 | Lack prevalence data |
| 285 | Myotonic dystrophy type I in childhood Long-term evolution in patients surviving the neonatal period | Echenne B | Eur J Paediatr Neurol | 2008 | Lack prevalence data |
| 286 | Seizure-genotype relationship in Fukuyama-type congenital muscular dystrophy | Yoshioka M | Brain Dev | 2008 | Lack prevalence data |
| 287 | Dynamin 2 mutations cause sporadic centronuclear myopathy with neonatal onset | Bitoun M | Ann Neurol | 2007 | Denominator/population is not clear |
| 288 | Refining genotype phenotype correlations in muscular dystrophies with defective glycosylation of dystroglycan | Godfrey C | Brain | 2007 | Denominator/population is not clear |
| 289 | Tuberculous myositis: an unusual presentation of extrapulmonary tuberculosis | Wang WY | J Microbiol Immunol Infect | 2007 | Denominator/population is not clear |
| 290 | Results of echocardiographic screening in 10,000 newborns | Wang NK | Acta Paediatr Taiwan | 2007 | Denominator/population is not clear |
| 291 | LGMD2I in a North American population | Kang PB | BMC Musculoskelet Disord | 2007 | Lack prevalence data |
| 292 | Abnormal sensorimotor integrative function of the larynx in congenital laryngomalacia: a new theory of etiology | Thompson DM | Laryngoscope | 2007 | Lack prevalence data |
| 293 | C-terminal titin deletions cause a novel early-onset myopathy with fatal cardiomyopathy | Carmignac V | Ann Neurol | 2007 | Lack prevalence data |
| 294 | Linburg-Comstock anomaly: seems to be harmless but may be fatal | Ortak T | Plast Reconstr Surg | 2007 | Lack prevalence data |
| 295 | Undetected central core disease myopathy in an infant presenting for clubfoot surgery | Zanette G | Paediatr Anaesth | 2007 | Lack prevalence data |
| 296 | Co-morbidity of Emery-Dreifuss muscular dystrophy and a congenital myasthenic syndrome possibly affecting the phenotype in a large Bedouin kindred | Ifergane G | Eur J Neurol | 2007 | Lack prevalence data |
| 297 | Congenital myotonic dystrophy: prenatal ultrasound findings and pregnancy outcome | Zaki M | Ultrasound Obstet Gynecol | 2007 | Lack prevalence data |
| 298 | Orofacial dysfunction in children and adolescents with myotonic dystrophy | Sjögreen L | Dev Med Child Neurol | 2007 | Lack prevalence data |
| 299 | Nemaline myopathy caused by absence of alpha-skeletal muscle actin | Nowak KJ | Ann Neurol | 2007 | Lack prevalence data |
| 300 | Clinical and echocardiographic features of primary infundibular stenosis with intact ventricular septum in dogs | Minors SL | J Vet Intern Med | 2006 | Lack prevalence data |
| 301 | Childhood acquired lipodystrophy: a retrospective study | Pope E | J Am Acad Dermatol | 2006 | Lack prevalence data |
| 302 | The relationship between developmental dysplasia of the hip and congenital muscular torticollis | von Heideken J | J Pediatr Orthop | 2006 | Lack prevalence data |
| 303 | A mutation in the fast skeletal muscle troponin I gene causes myopathy and distal arthrogryposis | Kimber E | Neurology | 2006 | Lack prevalence data |
| 304 | Congenital muscular dystrophy in Arab children | Habeeb YK | J Child Neurol | 2006 | Lack prevalence data |
| 305 | Familial risks for diseases of myoneural junction and muscle in siblings based on hospitalizations and deaths in sweden | Hemminki K | Twin Res Hum Genet | 2006 | Lack prevalence data |
| 306 | Lethal congenital contracture syndrome (LCCS) and other lethal arthrogryposes in Finland--an epidemiological study | Pakkasjärvi N | Am J Med Genet A | 2006 | Lack prevalence data |
| 307 | Atrial and ventricular septal defects - epidemiology and spontaneous closure | Garne E | J Matern Fetal Neonatal Med | 2006 | Lack prevalence data |
| 308 | Congenital contractural arachnodactyly (Beals syndrome) | Tunçbilek E | Orphanet J Rare Dis | 2006 | Lack prevalence data |
| 309 | Novel mutations in LAMA2 gene responsible for a severe phenotype of congenital muscular dystrophy in two Tunisian families | Louhichi N | Arch Inst Pasteur Tunis | 2006 | Lack prevalence data |
| 310 | Minicore myopathy with ophthalmoplegia caused by mutations in the ryanodine receptor type 1 gene | Jungbluth H | Neurology | 2005 | Denominator/population is not clear |
| 311 | Congenital muscular dystrophy with glycosylation defects of alpha-dystroglycan in Japan | Matsumoto H | Neuromuscul Disord | 2005 | Denominator/population is not clear |
| 312 | Subclinical cardiological involvement in Sicilian patients with pure congenital muscular dystrophy | Falsaperla R | Minerva Pediatr | 2005 | Lack prevalence data |
| 313 | Respiratory function assessment and intervention in neuromuscular disorders | Mellies U | Curr Opin Neurol | 2005 | Lack prevalence data |
| 314 | A multi-disciplinary study of the ocular, orthopedic, and neurologic causes of abnormal head postures in children | Nucci P | Am J Ophthalmol | 2005 | Lack prevalence data |
| 315 | Long-term prognosis of epilepsies and related seizure disorders in Fukuyama-type congenital muscular dystrophy | Yoshioka M | J Child Neurol | 2005 | Lack prevalence data |
| 316 | Elimination of myostatin does not combat muscular dystrophy in dy mice but increases postnatal lethality | Li ZF | Am J Pathol | 2005 | Lack prevalence data |
| 317 | Outcome in pregnancies complicated by myotonic dystrophy: a study of 31 patients and review of the literature | Rudnik-Schöneborn S | Eur J Obstet Gynecol Reprod Biol | 2004 | Denominator/population is not clear |
| 318 | Muscle disorders in pediatric patients in King Chulalongkorn Memorial Hospital | Jongpiputvanich S | J Med Assoc Thai | 2004 | Lack prevalence data |
| 319 | Evaluation of the inheritance of the complex vertebral malformation syndrome by breeding studies | Agerholm JS | Acta Vet Scand | 2004 | Lack prevalence data |
| 320 | Clinical and genetic heterogeneity of branching enzyme deficiency (glycogenosis type IV) | Bruno C | Neurology | 2004 | Lack prevalence data |
| 321 | Extended vertical trapezius myocutaneous flap in head and neck reconstruction as a salvage procedure | Uğurlu K | Plast Reconstr Surg | 2004 | Lack prevalence data |
| 322 | Congenital myotonic dystrophy: assisted ventilation duration and outcome | Campbell C | Pediatrics | 2004 | Lack prevalence data |
| 323 | Gene and cell-based therapies for heart disease | Melo LG | FASEB J | 2004 | Lack prevalence data |
| 324 | Congenital heart disease in infants of diabetic mothers: echocardiographic study | Abu-Sulaiman RM | Pediatr Cardiol | 2004 | Lack prevalence data |
| 325 | Worldwide distribution and broader clinical spectrum of muscle-eye-brain disease | Taniguchi K | Hum Mol Genet | 2003 | Denominator/population is not clear |
| 326 | The myopathology of floppy and hypotonic infants in Singapore | Premasiri MK | Pathology | 2003 | Lack prevalence data |
| 327 | Rapsyn mutations in hereditary myasthenia: distinct early- and late-onset phenotypes | Burke G | Neurology | 2003 | Lack prevalence data |
| 328 | The cnm locus, a canine homologue of human autosomal forms of centronuclear myopathy, maps to chromosome 2 | Tiret L | Hum Genet | 2003 | Lack prevalence data |
| 329 | Genetic counseling and prenatal diagnosis in India--experience at Sir Ganga Ram Hospital | Verma IC | Indian J Pediatr | 2003 | Lack prevalence data |
| 330 | Myotonic dystrophy type 2: molecular, diagnostic and clinical spectrum | Day JW | Neurology | 2003 | Lack prevalence data |
| 331 | The burden of genetic disorders in India and a framework for community control | Verma IC | Community Genet | 2002 | Lack prevalence data |
| 332 | Long-term outcome of congenital intestinal pseudoobstruction | Mousa H | Dig Dis Sci | 2002 | Lack prevalence data |
| 333 | Genetics of isolated and syndromic strabismus: facts and perspectives | Lorenz B | Strabismus | 2002 | Lack prevalence data |
| 334 | Mutations of the selenoprotein N gene, which is implicated in rigid spine muscular dystrophy, cause the classical phenotype of multiminicore disease: reassessing the nosology of early-onset myopathies | Ferreiro A | Am J Hum Genet | 2002 | Lack prevalence data |
| 335 | Congenital muscular dystrophy in Israeli families | Rachmiel M | J Child Neurol | 2002 | Lack prevalence data |
| 336 | The incidence of congenital heart disease | Hoffman JI | J Am Coll Cardiol | 2002 | Lack prevalence data |
| 337 | Myotonia dystrophica and spinal surgery | Colovic V | Paediatr Anaesth | 2002 | Lack prevalence data |
| 338 | Multiple congenital contractures: birth prevalence, etiology, and outcome | Darin N | J Pediatr | 2002 | Lack prevalence data |
| 339 | Nemaline myopathy: a clinical study of 143 cases | Ryan MM | Ann Neurol | 2001 | Denominator/population is not clear |
| 340 | Muscle CT in peripheral neuropathies | Marconi G | Acta Neurol Scand | 2001 | Lack prevalence data |
| 341 | Antiphospholipid antibodies and thrombophilic factors in giant cell arteritis | Espinosa G | Semin Arthritis Rheum | 2001 | Lack prevalence data |
| 342 | Diagnostic profile of neonatal hypotonia: an 11-year study | Richer LP | Pediatr Neurol | 2001 | Lack prevalence data |
| 343 | Etiology and outcome of hydrops fetalis | Ismail KM | J Matern Fetal Med | 2001 | Lack prevalence data |
| 344 | Enteric nervous system, interstitial cells of cajal, and smooth muscle vacuolization in segmental dilatation of jejunum | Cheng W | J Pediatr Surg | 2001 | Lack prevalence data |
| 345 | Fibrodysplasia ossificans progressiva | Mahboubi S | Pediatr Radiol | 2001 | Lack prevalence data |
| 346 | Pseudotumor of infancy and congenital muscular torticollis: 170 cases | Wei JL | Laryngoscope | 2001 | Lack prevalence data |
| 347 | Vacuolating megalencephalic leukoencephalopathy in 12 Israeli patients | Ben-Zeev B | J Child Neurol | 2001 | Lack prevalence data |
| 348 | MR evaluation of the hippocampus in patients with congenital malformations of the brain | Sato N | AJNR Am J Neuroradiol | 2001 | Lack prevalence data |
| 349 | Burden of genetic disorders in India | Verma IC | Indian J Pediatr | 2000 | Denominator/population is not clear |
| 350 | Age and origin of the FCMD 3'-untranslated-region retrotransposal insertion mutation causing Fukuyama-type congenital muscular dystrophy in the Japanese population | Colombo R | Hum Genet | 2000 | Denominator/population is not clear |
| 351 | Arthrogryposis, renal dysfunction and cholestasis syndrome | Abdullah MA | Saudi Med J | 2000 | Lack prevalence data |
| 352 | Biomechanical prenatal factors for the development of congenital hip dysplasia | Tarczyńska M | Ann Univ Mariae Curie Sklodowska Med | 2000 | Lack prevalence data |
| 353 | The congenitally missing upper lateral incisor. A retrospective study of orthodontic space closure versus restorative treatment | Robertsson S | Eur J Orthod | 2000 | Lack prevalence data |
| 354 | Isolated left ventricular abnormal trabeculation is a cardiac manifestation of neuromuscular disorders | Stöllberger C | Cardiology | 2000 | Lack prevalence data |
| 355 | Cardiac involvement in primary myopathies | Finsterer J | Cardiology | 2000 | Lack prevalence data |
| 356 | The expanding clinical and genetic spectrum of the myotonic dystrophies | Ricker K | Acta Neurol Belg | 2000 | Lack prevalence data |
| 357 | Scoliosis in arthrogryposis multiplex congenita: results after nonsurgical and surgical treatment | Yingsakmongkol W | J Pediatr Orthop | 2000 | Lack prevalence data |
| 358 | Proximal myotonic myopathy: evidence for anticipation in families with linkage to chromosome 3q | Schneider C | Neurology | 2000 | Lack prevalence data |
| 359 | Central core disease and congenital neuromuscular disease with uniform type 1 fibers in one family | Tojo M | Brain Dev | 2000 | Lack prevalence data |
| 360 | MTM1 mutations in X-linked myotubular myopathy | Laporte J | Hum Mutat | 2000 | Lack prevalence data |
| 361 | Epidemiology, presentation and management of congenital muscular torticollis | Ho BC | Singapore Med J | 1999 | Denominator/population is not clear |
| 362 | Adult onset reducing body myopathy | Figarella-Branger D | Neuromuscul Disord | 1999 | Lack prevalence data |
| 363 | Screening of the ryanodine receptor gene in 105 malignant hyperthermia families: novel mutations and concordance with the in vitro contracture test | Brandt A | Hum Mol Genet | 1999 | Lack prevalence data |
| 364 | Fibromatoses: clinical and pathological features suggestive of recurrence | Baerg J | J Pediatr Surg | 1999 | Lack prevalence data |
| 365 | Merosin-positive congenital muscular dystrophy: a large inbred family | Mahjneh I | Neuropediatrics | 1999 | Lack prevalence data |
| 366 | Isolated left ventricular abnormal trabeculation in adults is associated with neuromuscular disorders | Stöllberger C | Clin Cardiol | 1999 | Lack prevalence data |
| 367 | Prevalence of temporomandibular joint disk displacement in infants and young children | Paesani D | Oral Surg Oral Med Oral Pathol Oral Radiol Endod | 1999 | Lack prevalence data |
| 368 | Congenital muscular dystrophies: 1997 update | Voit T | Brain Dev | 1998 | Denominator/population is not clear |
| 369 | Neonatal screening for Duchenne muscular dystrophy: a novel semiquantitative application of the bioluminescence test for creatine kinase in a pilot national program in Cyprus | Drousiotou A | Genet Test | 1998 | Lack prevalence data |
| 370 | Myotonic dystrophy is a significant cause of idiopathic polyhydramnios | Esplin MS | Am J Obstet Gynecol | 1998 | Lack prevalence data |
| 371 | Diseases diagnosed in red foxes from the southeastern United States | Little SE | J Wildl Dis | 1998 | Lack prevalence data |
| 372 | Congenital abnormalities in Brazilian children associated with misoprostol misuse in first trimester of pregnancy | Gonzalez CH | Lancet | 1998 | Lack prevalence data |
| 373 | Immunohistochemical evaluation of merosin deficiency in congenital muscular dystrophies | Brett FM | Arch Pathol Lab Med | 1998 | Lack prevalence data |
| 374 | Genetic epidemiology of myotonic dystrophy in Istria, Croatia | Medica I | Acta Neurol Scand | 1997 | Denominator/population is not clear |
| 375 | Clinical and echocardiographic evaluation of neonates with heart murmurs | Du ZD | Acta Paediatr | 1997 | Lack prevalence data |
| 376 | Normal expression of adhalin and merosin in ovine congenital progressive muscular dystrophy | Johnsen RD | Aust Vet J | 1997 | Lack prevalence data |
| 377 | Limb swelling in patients who have fibrodysplasia ossificans progressiva | Moriatis JM | Clin Orthop Relat Res | 1997 | Lack prevalence data |
| 378 | Genetic epidemiology of congenital muscular dystrophy in a sample from north-east Italy | Mostacciuolo ML | Hum Genet | 1996 | Denominator/population is not clear |
| 379 | Childhood neuromuscular disorders: a decade's experience in Saudi Arabia | Salih MA | Ann Trop Paediatr | 1996 | Lack prevalence data |
| 380 | Linkage-disequilibrium mapping narrows the Fukuyama-type congenital muscular dystrophy (FCMD) candidate region to <100 kb | Toda T | Am J Hum Genet | 1996 | Lack prevalence data |
| 381 | Hydranencephaly, cerebellar hypoplasia, and myopathy in chick embryos infected with aino virus | Kitano Y | Vet Pathol | 1996 | Lack prevalence data |
| 382 | Deficiency of a skeletal muscle isoform of alpha-actinin (alpha-actinin-3) in merosin-positive congenital muscular dystrophy | North KN | Neuromuscul Disord | 1996 | Lack prevalence data |
| 383 | Congenital muscular dystrophy with cerebral white matter hypodensity. Correlation of clinical features and merosin deficiency | Reed UC | Brain Dev | 1996 | Lack prevalence data |
| 384 | Hereditary disorders among Iranian Jews | Zlotogora J | Am J Med Genet | 1995 | Lack prevalence data |
| 385 | A gene for a severe lethal form of X-linked arthrogryposis (X-linked infantile spinal muscular atrophy) maps to human chromosome Xp11.3-q11.2 | Kobayashi H | Hum Mol Genet | 1995 | Lack prevalence data |
| 386 | X linked fatal infantile cardiomyopathy maps to Xq28 and is possibly allelic to Barth syndrome | Gedeon AK | J Med Genet | 1995 | Lack prevalence data |
| 387 | Myotonic dystrophy: genetic, clinical, and molecular analysis of patients from 41 Brazilian families | Passos-Bueno MR | J Med Genet | 1995 | Lack prevalence data |
| 388 | Prevalence of unsuspected myopathy in infants presenting for clubfoot surgery | Zanette G | Paediatr Anaesth | 1995 | Lack prevalence data |
| 389 | Presentation, clinical course, and outcome of the congenital form of myotonic dystrophy | Roig M | Pediatr Neurol | 1994 | Lack prevalence data |
| 390 | Severe complications of the anterior cricoid split operation and single-stage laryngotracheoplasty | Zeitouni AG | Ann Otol Rhinol Laryngol | 1994 | Lack prevalence data |
| 391 | Cardiomyopathy in childhood and adult life, with emphasis on hypertrophic cardiomyopathy | Landing BH | Pathol Res Pract | 1994 | Lack prevalence data |
| 392 | Non-Mendelian mitochondrial inheritance as a cause of progressive genetic sensorineural hearing loss | Gold M | Int J Pediatr Otorhinolaryngol | 1994 | Lack prevalence data |
| 393 | Anticipation resulting in elimination of the myotonic dystrophy gene: a follow up study of one extended family | de Die-Smulders CE | J Med Genet | 1994 | Lack prevalence data |
| 394 | Lethal congenital contracture syndrome: further delineation and genetic aspects | Vuopala K | J Med Genet | 1994 | Lack prevalence data |
| 395 | Multicore myopathy, microcephaly, aganglionosis, and short stature | Kim JJ | J Child Neurol | 1994 | Lack prevalence data |
| 396 | Pigmented villonodular synovitis in a child | Soifer T | J Pediatr Surg | 1993 | Lack prevalence data |
| 397 | Indications and contraindications for heart transplantation in infancy | Boucek MM | J Heart Lung Transplant | 1993 | Lack prevalence data |
| 398 | The role of the dystrophin-glycoprotein complex in the molecular pathogenesis of muscular dystrophies | Matsumura K | Neuromuscul Disord | 1993 | Lack prevalence data |
| 399 | Chronic ventilator-assisted children in university hospitals in Japan | Sakakihara Y | Acta Paediatr Jpn | 1993 | Lack prevalence data |
| 400 | The frequency of intradural conjoined lumbosacral dorsal nerve roots found during selective dorsal rhizotomy | Phillips LH 2nd | Neurosurgery | 1993 | Lack prevalence data |
| 401 | Respiratory involvement in primary muscle disorders: assessment and management | Howard RS | Q J Med | 1993 | Lack prevalence data |
| 402 | The natural history of congenital myotonic dystrophy: mortality and long term clinical aspects | Reardon W | Arch Dis Child | 1993 | Lack prevalence data |
| 403 | An outbreak of Akabane virus-induced abnormalities in calves after agistment in an endemic region | Jagoe S | Aust Vet J | 1993 | Lack prevalence data |
| 404 | Explanation for exclusive maternal origin for congenital form of myotonic dystrophy | Mulley JC | Lancet | 1993 | Lack prevalence data |
| 405 | A community survey of neurological disorders in Saudi Arabia: the Thugbah study | al Rajeh S | Neuroepidemiology | 1993 | Lack prevalence data |
| 406 | Clinical features for prediction of survival in neonatal muscle disease | Connolly MB | Pediatr Neurol | 1992 | Lack prevalence data |
| 407 | A large inbred Palestinian family with two forms of muscular dystrophy | Mahjneh I | Neuromuscul Disord | 1992 | Lack prevalence data |
| 408 | Epidemiology of neuromuscular disorders in the under-20 population in Bologna Province, Italy | Merlini L | Neuromuscul Disord | 1992 | Lack prevalence data |
| 409 | Clinico-pathological analysis of foal diseases from 237 autopsy cases | Oikawa MA | Kitasato Arch Exp Med | 1991 | Denominator/population is not clear |
| 410 | Dyserythropoiesis, polymyopathy, and cardiac disease in three related English springer spaniels | Holland CT | J Vet Intern Med | 1991 | Denominator/population is not clear |
| 411 | MR imaging of the brain in Fukuyama-type congenital muscular dystrophy | Yoshioka M | AJNR Am J Neuroradiol | 1991 | Denominator/population is not clear |
| 412 | The management of trigger thumb in children | Ger E | J Hand Surg Am | 1991 | Lack prevalence data |
| 413 | Glyceroluria in healthy adults, mentally ill adults and children selected for metabolic screening | Kohlschütter A | Clin Chim Acta | 1991 | Lack prevalence data |
| 414 | Fukuyama type congenital progressive muscular dystrophy | Osawa M | Acta Paediatr Jpn | 1991 | Lack prevalence data |
| 415 | Population frequencies of inherited neuromuscular diseases--a world survey | Emery AE | Neuromuscul Disord | 1991 | Lack prevalence data |
| 416 | Epidemiology of progressive muscular dystrophy in Okinawa, Japan. Classification with molecular biological techniques | Nakagawa M | Neuroepidemiology | 1991 | Lack prevalence data |
| 417 | Fukuyama type congenital muscular dystrophy in a Turkish child | Topaloğlu H | Can J Neurol Sci | 1990 | Lack prevalence data |
| 418 | Evidence that Cache Valley virus induces congenital malformations in sheep | Chung SI | Vet Microbiol | 1990 | Lack prevalence data |
| 419 | Histopathologic study on muscle diseases among Koreans (274 muscle biopsy analysis) | Chi JG | J Korean Med Sci | 1989 | Lack prevalence data |
| 420 | Congenital myotonic dystrophy: respiratory function at birth determines survival | Rutherford MA | Arch Dis Child | 1989 | Lack prevalence data |
| 421 | Congenital muscular dystrophy (non-Fukuyama type) in Turkey: a clinical and pathological evaluation | Topaloğlu H | Brain Dev | 1989 | Lack prevalence data |
| 422 | Cerebro-ocular-muscular syndrome in a Portuguese family | Leão M | Prog Clin Biol Res | 1989 | Lack prevalence data |
| 423 | Ovine arthrogryposis and central nervous system malformations associated with in utero Cache Valley virus infection: spontaneous disease | Edwards JF | Vet Pathol | 1989 | Lack prevalence data |
| 424 | Laboratory investigation of a naturally occurring outbreak of arthrogryposis-hydranencephaly in Texas sheep | Crandell RA | J Vet Diagn Invest | 1989 | Lack prevalence data |
| 425 | The development of Akabane virus-induced congenital abnormalities in cattle | Kirkland PD | Vet Rec | 1988 | Lack prevalence data |
| 426 | Comparative study of neurological and myxoedematous cretinism associated with severe iodine deficiency | Chaouki ML | Clin Endocrinol (Oxf) | 1988 | Lack prevalence data |
| 427 | Immaturity of muscle fibers in the congenital form of myotonic dystrophy: its consequences and its origin | Farkas-Bargeton E | J Neurol Sci | 1988 | Lack prevalence data |
| 428 | Neonatal myotonic dystrophy | Cunningham A | Aust Paediatr J | 1988 | Lack prevalence data |
| 429 | Report of the delegation of clinical geneticists to China, Spring 1986 | Laurence M | Biol Soc | 1987 | Denominator/population is not clear |
| 430 | Type 1 fiber size disproportion: morphometric data from 37 children with myopathic, neuropathic, or idiopathic hypotonia | Iannaccone ST | Pediatr Pathol | 1987 | Denominator/population is not clear |
| 431 | Congenital myotonic dystrophy. Incidence, clinical aspects and early prognosis | Wesström G | Acta Paediatr Scand | 1986 | Lack prevalence data |
| 432 | A variant of Fukuyama congenital muscular dystrophy in a non-Japanese child | Kohrman MH | Pediatr Neurol | 1986 | Lack prevalence data |
| 433 | Frequency of various congenital heart diseases in Chinese adults: analysis of 926 consecutive patients over 13 years of age | Lien WP | Am J Cardiol | 1986 | Lack prevalence data |
| 434 | Multiple congenital malformations of the face, nervous system and musculoskeletal system of pigs | Whittington RJ | Aust Vet J | 1986 | Lack prevalence data |
| 435 | Pleocore disease. Multi-minicore disease and focal loss of cross striations | Martin JJ | Acta Neuropathol | 1986 | Lack prevalence data |
| 436 | Muscle diseases in Singapore | Lee YS | Pathology | 1986 | Lack prevalence data |
| 437 | The genetics of muscular dystrophies | Harper PS | Prog Med Genet | 1985 | Lack prevalence data |
| 438 | Congenital, latent and manifest latent nystagmus--similarities, differences and relation to strabismus | Dell'Osso LF | Jpn J Ophthalmol | 1985 | Lack prevalence data |
| 439 | Associations of sire, breed, birth weight, and sex in pigs with congenital splayleg | Vogt DW | Am J Vet Res | 1984 | Lack prevalence data |
| 440 | Congenital muscular dystrophy, brain malformation and ocular problems (muscle, eye and brain disease) in two German families | Korinthenberg R | Eur J Pediatr | 1984 | Lack prevalence data |
| 441 | Posttraumatic sports-related musculoskeletal abnormalities: prevalence in a normal population | Raskin RJ | Am J Sports Med | 1983 | Denominator/population is not clear |
| 442 | Early infant death in nemaline (rod) myopathy | Eeg-Olofsson O | Brain Dev | 1983 | Denominator/population is not clear |
| 443 | An impending epidemic of bovine congenital deformities | Kirkland PD | Aust Vet J | 1983 | Lack prevalence data |
| 444 | X-linked dominant inherited diseases with lethality in hemizygous males | Wettke-Schäfer R | Hum Genet | 1983 | Lack prevalence data |
| 445 | Fukuyama-type congenital muscular dystrophy | McMenamin JB | J Pediatr | 1982 | Lack prevalence data |
| 446 | Akabane disease in cattle: congenital abnormalities caused by viral infection. Spontaneous disease | Konno S | Vet Pathol | 1982 | Lack prevalence data |
| 447 | Comparative study of hospital incidences of congenital anomalies, childhood malignant solid tumours and pyomyositis in mainland Tanzania | Shija JK | Trop Doct | 1981 | Lack prevalence data |
| 448 | The 1960s epidemic of arthrogryposis multiplex congenita: a survey from the United Kingdom, Australia and the United States of America | Wynne-Davies R | J Bone Joint Surg Br | 1981 | Lack prevalence data |
| 449 | Diverticular disease of the colon in a far-eastern community | Vajrabukka T | Dis Colon Rectum | 1980 | Lack prevalence data |
| 450 | Congenital bovine epizootic arthrogryposis and hydranencephaly | Shepherd NC | Aust Vet J | 1978 | Lack prevalence data |
| 451 | Survey of Duchenne type and congenital type of muscular dystrophy in Shimane, Japan | Takeshita K | Jinrui Idengaku Zasshi | 1977 | Lack prevalence data |
| 452 | Congenital bovine epizootic arthrogryposis and hydranencephaly in Australia. Distribution of antibodies to Akabane virus in Australian Cattle after the 1974 epizootic | Della-Porta AJ | Aust Vet J | 1976 | Lack prevalence data |
| 453 | Arthrogryposis multiplex congenita. Search for prenatal factors in 66 sporadic cases | Wynne-Davies R | Arch Dis Child | 1976 | Lack prevalence data |
| 454 | Electromyographic findings in the so-called non-progressive myopathies | Hausmanowa-Petrusewicz I | J Neurol | 1976 | Lack prevalence data |
| 455 | Congenital myotonic dystrophy in Britain. II. Genetic basis | Harper PS | Arch Dis Child | 1975 | Lack prevalence data |
| 456 | Congenital dislocation of the hip associated with central core disease | Ramsey PL | J Bone Joint Surg Am | 1975 | Lack prevalence data |
| 457 | Congenital myotonic dystrophy in Britain. I. Clinical aspects | Harper PS | Arch Dis Child | 1975 | Lack prevalence data |
| 458 | Perosomus elumbis in sheep | Dennis SM | Aust Vet J | 1975 | Lack prevalence data |
| 459 | The prognostic implications of suppression-burst activity in the EEG in infancy | Maheshwari MC | Epilepsia | 1975 | Lack prevalence data |
| 460 | Hypotonia in the blind child | Jan JE | Dev Med Child Neurol | 1975 | Lack prevalence data |
| 461 | Congenital hypoplasia of depressor anguli oris muscle. A genetically determined condition? | Papadatos C | Arch Dis Child | 1974 | Denominator/population is not clear |
| 462 | Congenital and hereditary abnormalities in the elderly | Haleem MA | Gerontol Clin (Basel) | 1974 | Lack prevalence data |
| 463 | Genetic disorders in isolated populations | Scott EM | Arch Environ Health | 1973 | Lack prevalence data |
| 464 | Congenital defects of cattle: nature, cause, and effect | Leipold HW | Adv Vet Sci Comp Med | 1972 | Lack prevalence data |
| 465 | Respiratory syncytial virus in hospital cross-infection | Ditchburn RK | Br Med J | 1971 | Lack prevalence data |
| 466 | Arthrogryposis multiplex congenita | Gibson DA | J Bone Joint Surg Br | 1970 | Lack prevalence data |
| 467 | Childhood cancer and congenital defects. A study of U.S. death certificates during the period 1960-1966 | Miller RW | Pediatr Res | 1969 | Denominator/population is not clear |
| 468 | Congenital malformations of the cerebrospinal axis seen in Western Nigeria. The spinal meningoceles | Odeku EL | Int Surg | 1967 | Lack prevalence data |

**Supplementary Table S2. Quality of study reporting assessment**

| Study | Description of study design and setting | Description of eligibility criteria | Study population | Description of outcomes | Description of the study participants | Overall score |
| --- | --- | --- | --- | --- | --- | --- |
| Amburgey 2011 | Yes | Yes | Yes | No | No | Medium |
| Chung 2003 | Yes | Yes | Yes | No | No | Medium |
| Darin 2000 | Yes | Yes | Yes | No | No | Medium |
| Hughes 1996 | Yes | Yes | Yes | No | No | Medium |
| Lefter 2017 | Yes | Yes | Yes | No | No | Medium |
| Norwood 2009 | Yes | Yes | Yes | No | No | Medium |
| Pagola-Lorz 2019 | Yes | Yes | Yes | No | No | Medium |
| Santos 2006 | No | No | Yes | No | No | Low |
| Tangsrud 1988 | Yes | Yes | Yes | No | No | Medium |
| Theadom 2019 | Yes | Yes | Yes | No | No | Medium |
| Witting 2017 | Yes | Yes | Yes | No | No | Medium |


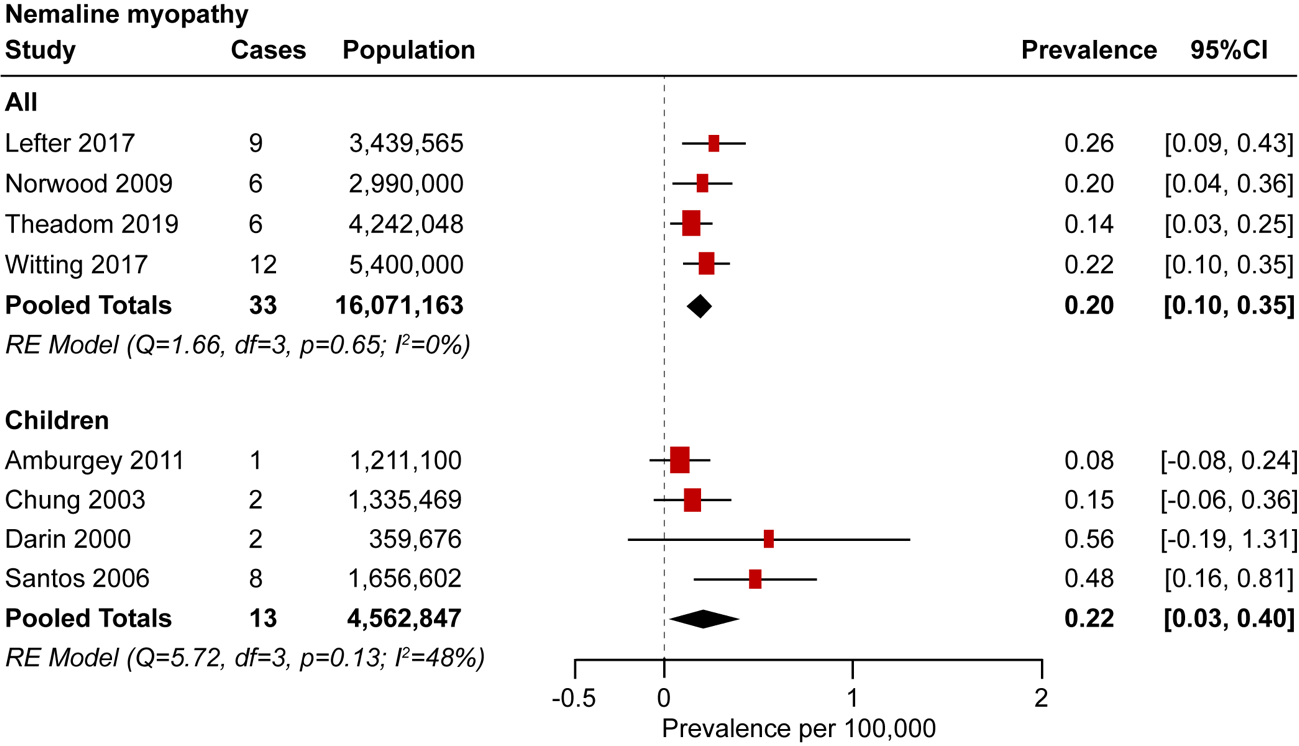


**Supplementary Figure S1.** Forest plot of the estimated prevalence of nemaline myopathy per 100,000 cases along with 95% confidence interval (CI).


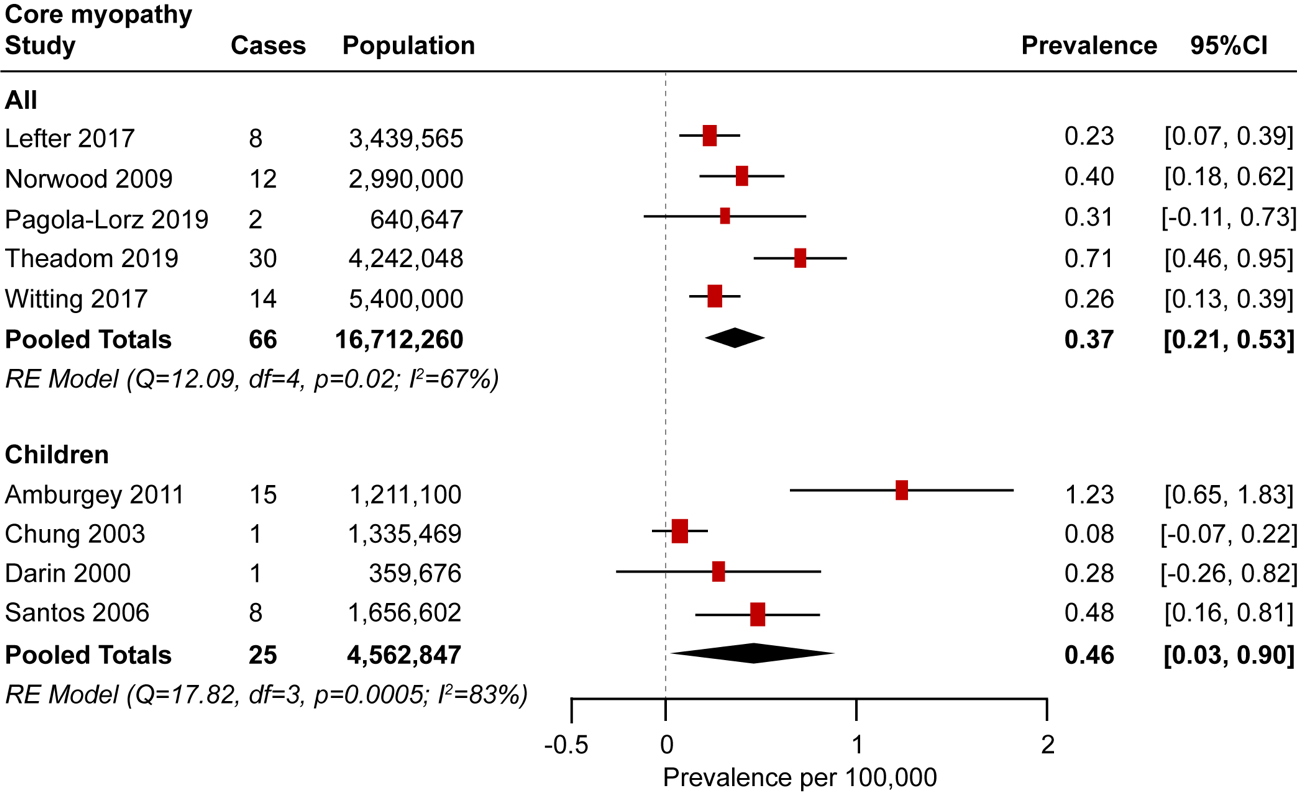


**Supplementary Figure S2.** Forest plot of the estimated prevalence of core myopathy per 100,000 cases along with the 95% confidence interval (CI).

**
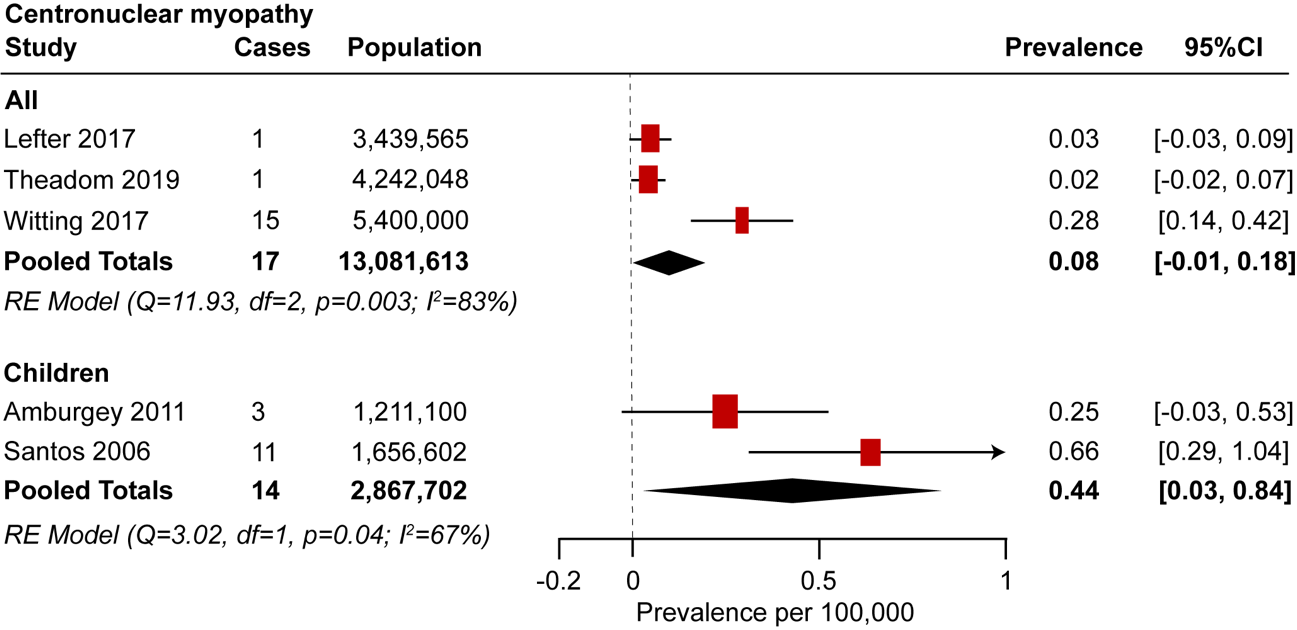
**

**Supplementary Figure S3.** Forest plot of the estimated prevalence of centronuclear myopathy per 100,000 cases along with the 95% confidence interval (CI).

**
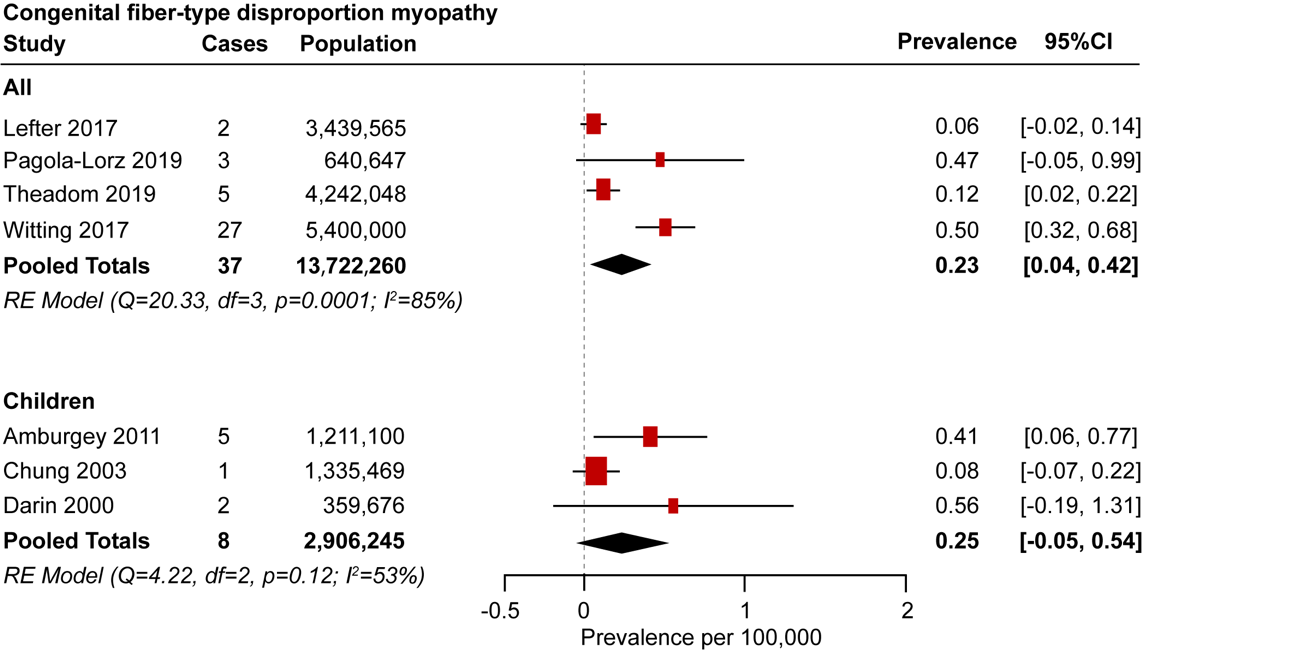
**

**Supplementary Figure S4.** Forest plot of the estimated prevalence of congenital fiber-type disproportion myopathy per 100,000 cases along with the 95% confidence interval (CI).

**
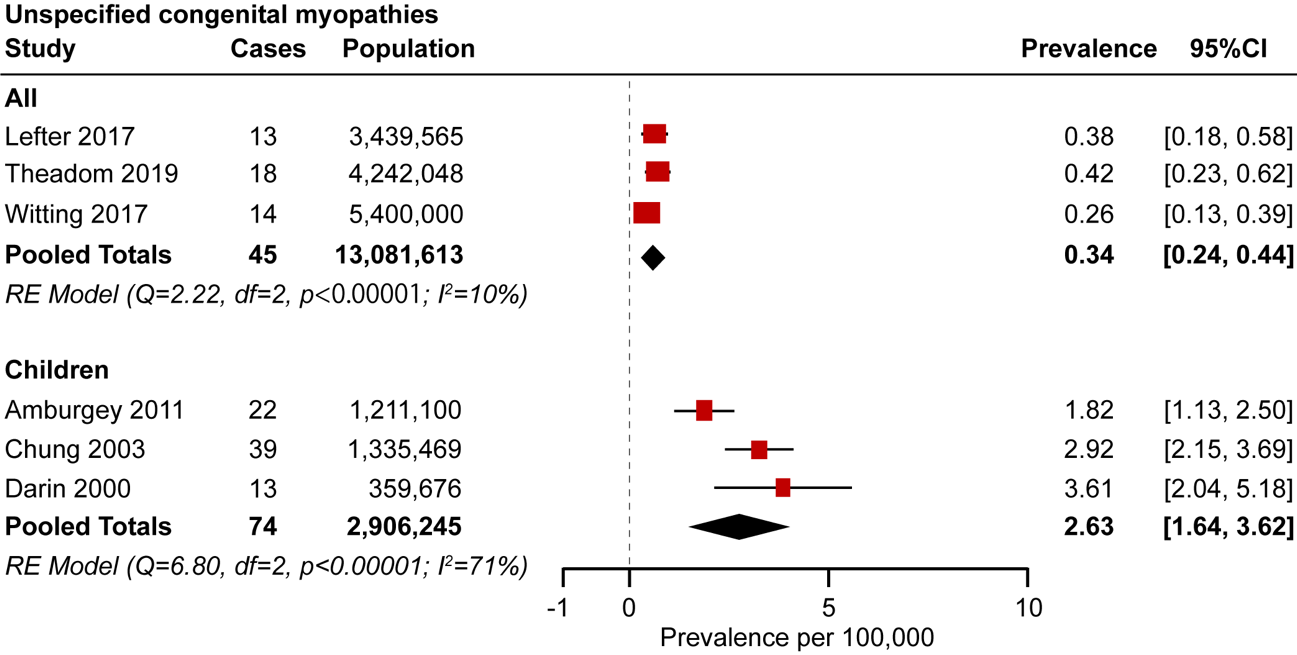
**

**Supplementary Figure S5.** Forest plot of the estimated prevalence of unspecified congenital myopathies per 100,000 cases along with the 95% confidence interval (CI).
